# Supplementary material for: PrimPol Is Required for Replicative Tolerance of G Quadruplexes in Vertebrate Cells
Source: Mol Cell. 2016 Jan 7;61(1):161–9. doi: 10.1016/j.molcel.2015.10.038 (PMC4712188; doi:10.1016/j.molcel.2015.10.038)
Supplement: Document S1. Supplemental Experimental Procedures and Figures S1–S4 [file mmc1.pdf]

Molecular Cell

Supplemental Information

PrimPol Is Required for Replicative Tolerance of G Quadruplexes in Vertebrate Cells

Davide Schiavone, Stanislaw K. Jozwiakowski, Marina Romanello, Guillaume Guilbaud, Thomas A. Guilliam, Laura J. Bailey, Julian E. Sale, and Aidan J. Doherty

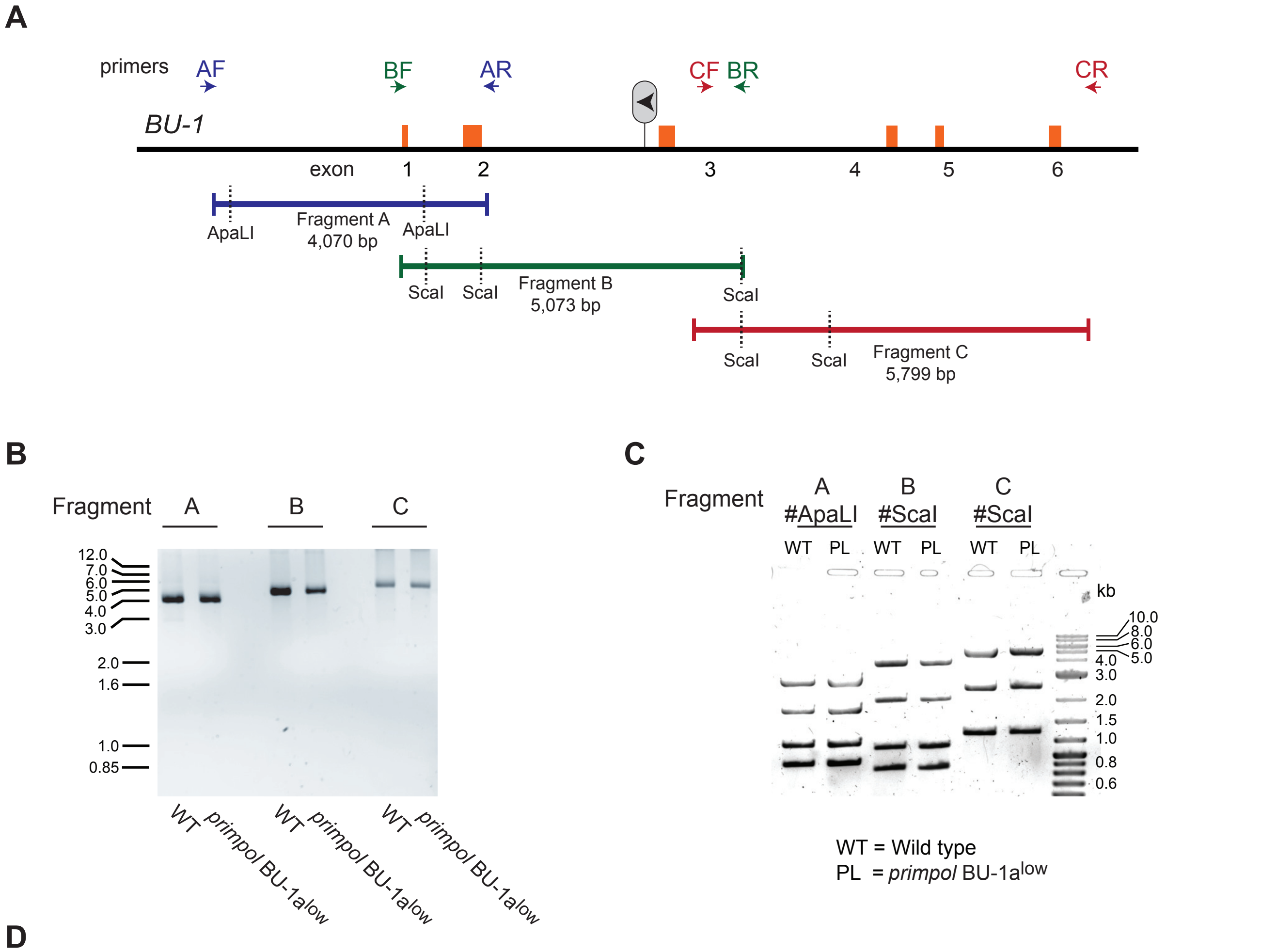

D

```
DT40 Bu1a          1 GTGTCTGGCGTTGACACCTACCTTGAGCATGGTTAGTACCAATTCCTCTCTAAAACAGAAGCAGATACAGTAACAGCAAAAGTACAGACCTCTGGCCAACGAGAAGGCTGACTCTCCTCTGAAGCTATAA 130
Primpol Bu1 low    1 GTGTCTGGCGTTGACACCTACCTTGAGCATGGTTAGTACCAATTCCTCTCTAAAACAGAAGCAGATACAGTAACAGCAAAAGTACAGACCTCTGGCCAACGAGAAGGCTGACTCTCCTCTGAAGCTATAA 130
Primpol Bu1 low    1 GTGTCTGGCGTTGACACCTACCTTGAGCATGGTTAGTACCAATTCCTCTCTAAAACAGAAGCAGATACAGTAACAGCAAAAGTACAGACCTCTGGCCAACGAGAAGGCTGACTCTCCTCTGAAGCTATAA 130
Primpol Bu1 low    1 GTGTCTGGCGTTGACACCTACCTTGAGCATGGTTAGTACCAATTCCTCTCTAAAACAGAAGCAGATACAGTAACAGCAAAAGTACAGACCTCTGGCCAACGAGAAGGCTGACTCTCCTCTGAAGCTATAA 130
Primpol Bu1 low    1 GTGTCTGGCGTTGACACCTACCTTGAGCATGGTTAGTACCAATTCCTCTCTAAAACAGAAGCAGATACAGTAACAGCAAAAGTACAGACCTCTGGCCAACGAGAAGGCTGACTCTCCTCTGAAGCTATAA 130
Primpol Bu1 low    1 GTGTCTGGCGTTGACACCTACCTTGAGCATGGTTAGTACCAATTCCTCTCTAAAACAGAAGCAGATACAGTAACAGCAAAAGTACAGACCTCTGGCCAACGAGAAGGCTGACTCTCCTCTGAAGCTATAA 130
Primpol Bu1 low    1 GTGTCTGGCGTTGACACCTACCTTGAGCATGGTTAGTACCAATTCCTCTCTAAAACAGAAGCAGATACAGTAACAGCAAAAGTACAGACCTCTGGCCAACGAGAAGGCTGACTCTCCTCTGAAGCTATAA 130
Primpol Bu1 low    1 GTGTCTGGCGTTGACACCTACCTTGAGCATGGTTAGTACCAATTCCTCTCTAAAACAGAAGCAGATACAGTAACAGCAAAAGTACAGACCTCTGGCCAACCAGAAGGCTGACTCTCCTCTGAAGCTATAA 130
Primpol Bu1 low    1 GTGTCTGGCGTTGACACCTACCTTGAGCATGGTTAGTACCAATTCCTCTCTAAAACAGAAGCAGATACAGTAACAGCAAAAGTACAGACCTCTGGCCAACCAGAAGGCTGACTCTCCTCTGAAGCTATAA 130
Primpol Bu1 low    1 GTGTCTGGCGTTGACACCTACCTTGAGCATGGTTAGTACCAATTCCTCTCTAAAACAGAAGCAGATACAGTAACAGCAAAAGTACAGACCTCTGGCCAACCAGAAGGCTGACTCTCCTCTGAAGCTATAA 130
                     **************************************************************************************************** *****************************

DT40 Bu1a        131 TGGGTATAATCAATTACCTATTCATTATATAGGAAGCCTCTGGCCAGCAGAAGCTTAGCGTGCCCTCAGATGATGGTCTTCTGTTGCTATCATTTTCACTTTCAACAAGGAAAATAAAGAGGTGCCATAT 260
Primpol Bu1 low  131 TGGGTATAATCAATTACCTATTCATTATATAGGAAGCCTCTGGCCAGCAGAAGCTTAGCGTGCCCTCAGATGATGGTCTTCTGTTGCTATCATTTTCACTTTCAACAAGGAAAATAAAGAGGTGCCATAT 260
Primpol Bu1 low  131 TGGGTATAATCAATTACCTATTCATTATATAGGAAGCCTCTGGCCAGCAGAAGCTTAGCGTGCCCTCAGATGATGGTCTTCTGTTGCTATCATTTTCACTTTCAACAAGGAAAATAAAGAGGTGCCATAT 260
Primpol Bu1 low  131 TGGGTATAATCAATTACCTATTCATTATATAGGAAGCCTCTGGCCAGCAGAAGCTTAGCGTGCCCTCAGATGATGGTCTTCTGTTGCTATCATTTTCACTTTCAACAAGGAAAATAAAGAGGTGCCATAT 260
Primpol Bu1 low  131 TGGGTATAATCAATTACCTATTCATTATATAGGAAGCCTCTGGCCAGCAGAAGCTTAGCGTGCCCTCAGATGATGGTCTTCTGTTGCTATCATTTTCACTTTCAACAAGGAAAATAAAGAGGTGCCATAT 260
Primpol Bu1 low  131 TGGGTATAATCAATTACCTATTCATTATATAGGAAGCCTCTGGCCAGCAGAAGCTTAGCGTGCCCTCAGATGATGGTCTTCTGTTGCTATCATTTTCACTTTCAACAAGGAAAATAAAGAGGTGCCATAT 260
Primpol Bu1 low  131 TGGGTATAATCAATTACCTATTCATTATATAGGAAGCCTCTGGCCAGCAGAAGCTTAGCGTGCCCTCAGATGATGGTCTTCTGTTGCTATCATTTTCACTTTCAACAAGGAAAATAAAGAGGTGCCATAT 260
Primpol Bu1 low  131 TGGGTATAATCAATTACCTATTCATTATATAGGAAGCCTCTGGCCAGCAGAAGCTTAGCGTGCCCTCAGATGATGGTCTTCTGTTGCTATCATTTTCACTTTCAACAAGGAAAATAAAGAGGTGCCATAT 260
Primpol Bu1 low  131 TGGGTATAATCAATTACCTATTCATTATATAGGAAGCCTCTGGCCAGCAGAAGCTTAGCGTGCCCTCAGATGATGGTCTTCTGTTGCTATCATTTTCACTTTCAACAAGGAAAATAAAGAGGTGCCATAT 260
Primpol Bu1 low  131 TGGGTATAATCAATTACCTATTCATTATATAGGAAGCCTCTGGCCAGCAGAAGCTTAGCGTGCCCTCAGATGATGGTCTTCTGTTGCTATCATTTTCACTTTCAACAAGGAAAATAAAGAGGTGCCATAT 260
                     **********************************************************************************************************************************

DT40 Bu1a        261 TTTTAGGGTGTCCCCAAAATGTTGACCAAGTGTCACCAGTAGGGATGGGTGGGTGCTGTCAAGGGCTGGGATCTAGCAATTGAAGAGTCTGGTTACTTAGTGATGTGCTCCCTGGCTAACAGTGAATGTG 390
Primpol Bu1 low  261 TTTTAGGGTGTCCCCAAAATGTTGACCAAGTGTCACCAGTAGGGATGGGTGGGTGCTGTCAAGGGCTGGGATCTAGCAATTGAAGAGTCTGGTTACTTAGTGATGTGCTCCCTGGCTAACAGTGAATGTG 390
Primpol Bu1 low  261 TTTTAGGGTGTCCCCAAAATGTTGACCAAGTGTCACCAGTAGGGATGGGTGGGTGCTGTCAAGGGCTGGGATCTAGCAATTGAAGAGTCTGGTTACTTAGTGATGTGCTCCCTGGCTAACAGTGAATGTG 390
Primpol Bu1 low  261 TTTTAGGGTGTCCCCAAAATGTTGACCAAGTGTCACCAGTAGGGATGGGTGGGTGCTGTCAAGGGCTGGGATCTAGCAATTGAAGAGTCTGGTTACTTAGTGATGTGCTCCCTGGCTAACAGTGAATGTG 390
Primpol Bu1 low  261 TTTTAGGGTGTCCCCAAAATGTTGACCAAGTGTCACCAGTAGGGATGGGTGGGTGCTGTCAAGGGCTGGGATCTAGCAATTGAAGAGTCTGGTTACTTAGTGATGTGCTCCCTGGCTAACAGTGAATGTG 390
Primpol Bu1 low  261 TTTTAGGGTGTCCCCAAAATGTTGACCAAGTGTCACCAGTAGGGATGGGTGGGTGCTGTCAAGGGCTGGGATCTAGCAATTGAAGAGTCTGGTTACTTAGTGATGTGCTCCCTGGCTAACAGTGAATGTG 390
Primpol Bu1 low  261 TTTTAGGGTGTCCCCAAAATGTTGACCAAGTGTCACCAGTAGGGATGGGTGGGTGCTGTCAAGGGCTGGGATCTAGCAATTGAAGAGTCTGGTTACTTAGTGATGTGCTCCCTGGCTAACAGTGAATGTG 390
Primpol Bu1 low  261 TTTTAGGGTGTCCCCAAAATGTTGACCAAGTGTCACCAGTAGGGATGGGTGGGTGCTGTCAAGGGCTGGGATCTAGCAATTGAAGAGTCTGGTTACTTAGTGATGTGCTCCCTGGCTAACAGTGAATGTG 390
Primpol Bu1 low  261 TTTTAGGGTGTCCCCAAAATGTTGACCAAGTGTCACCAGTAGGGATGGGTGGGTGCTGTCAAGGGCTGGGATCTAGCAATTGAAGAGTCTGGTTACTTAGTGATGTGCTCCCTGGCTAACAGTGAATGTG 390
Primpol Bu1 low  261 TTTTAGGGTGTCCCCAAAATGTTGACCAAGTGTCACCAGTAGGGATGGGTGGGTGCTGTCAAGGGCTGGGATCTAGCAATTGAAGAGTCTGGTTACTTAGTGATGTGCTCCCTGGCTAACAGTGAATGTG 390
                     **********************************************************************************************************************************
                                                                 +3.5 G4

DT40 Bu1a        391 GTGGGAGCCCTCCTGGTAGCGGGGAGCTCTCAGTTACTGCTGAGTCTGCTCATCATCTCTCTTCCCTCTCCATTCCCCATTGCCCTGTGCTACGTGACCAGAGCCGATCATTGATGCCCGG 511
Primpol Bu1 low  391 GTGGGAGCCCTCCTGGTAGCGGGGAGCTCTCAGTTACTGCTGAGTCTGCTCATCATCTCTCTTCCCTCTCCATTCCCCATTGCCCTGTGCTACGTGACCAGAGCCGATCATTGATGCCCGG 511
Primpol Bu1 low  391 GTGGGAGCCCTCCTGGTAGCGGGGAGCTCTCAGTTACTGCTGAGTCTGCTCATCATCTCTCTTCCCTCTCCATTCCCCATTGCCCTGTGCTACGTGACCAGAGCCGATCATTGATGCCCGG 511
Primpol Bu1 low  391 GTGGGAGCCCTCCTGGTAGCGGGGAGCTCTCAGTTACTGCTGAGTCTGCTCATCATCTCTCTTCCCTCTCCATTCCCCATTGCCCTGTGCTACGTGACCAGAGCCGATCATTGATGCCCGG 511
Primpol Bu1 low  391 GTGGGAGCCCTCCTGGTAGCGGGGAGCTCTCAGTTACTGCTGAGTCTGCTCATCATCTCTCTTCCCTCTCCATTCCCCATTGCCCTGTGCTACGTGACCAGAGCCGATCATTGATGCCCGG 511
Primpol Bu1 low  391 GTGGGAGCCCTCCTGGTAGCGGGGAGCTCTCAGTTACTGCTGAGTCTGCTCATCATCTCTCTTCCCTCTCCATTCCCCATTGCCCTGTGCTACGTGACCAGAGCCGATCATTGATGCCCGG 511
Primpol Bu1 low  391 GTGGGAGCCCTCCTGGTAGCGGGGAGCTCTCAGTTACTGCTGAGTCTGCTCATCATCTCTCTTCCCTCTCCATTCCCCATTGCCCTGTGCTACGTGACCAGAGCCGATCATTGATGCCCGG 511
Primpol Bu1 low  391 GTGGGAGCCCTCCTGGTAGCGGGGAGCTCTCAGTTACTGCTGAGTCTGCTCATCATCTCTCTTCCCTCTCCATTCCCCATTGCCCTGTGCTACGTGACCAGAGCCGATCATTGATGCCCGG 511
Primpol Bu1 low  391 GTGGGAGCCCTCCTGGTAGCGGGGAGCTCTCAGTTACTGCTGAGTCTGCTCATCATCTCTCTTCCCTCTCCATTCCCCATTGCCCTGTGCTACGTGACCAGAGCCGATCATTGATGCCCGG 511
Primpol Bu1 low  391 GTGGGAGCCCTCCTGGTAGCGGGGAGCTCTCAGTTACTGCTGAGTCTGCTCATCATCTCTCTTCCCTCTCCATTCCCCATTGCCCTGTGCTACGTGACCAGAGCCGATCATTGATGCCCGG 511
                     *************************************************************************************************************************
```

Supplementary Figures

Figure S1. No evidence of high levels of mutation or genetic instability in the *BU-1* locus of Bu-1a^{low} cells. Related to Figure 1.

A. Diagram of the *BU-1* locus indicating PCR fragments A, B & C used to detect gross sequence deletions and the relevant restriction enzyme cutting sites in the fragments.

B. PCR amplification of fragments A, B & C from wild type cells and a pool of sorted Bu-1a^{low} cells.

C. Restriction digestion of the fragments shown in (B) cloned into pBluescript.

D. Sequences from 9 clones taken from around the +3.5 G4 (highlighted in bold). A polymorphism identifies the *BU-1A* allele (red) and *BU-1B* allele (blue).

Schiavone, Jozwiakowski et al. Figure S2

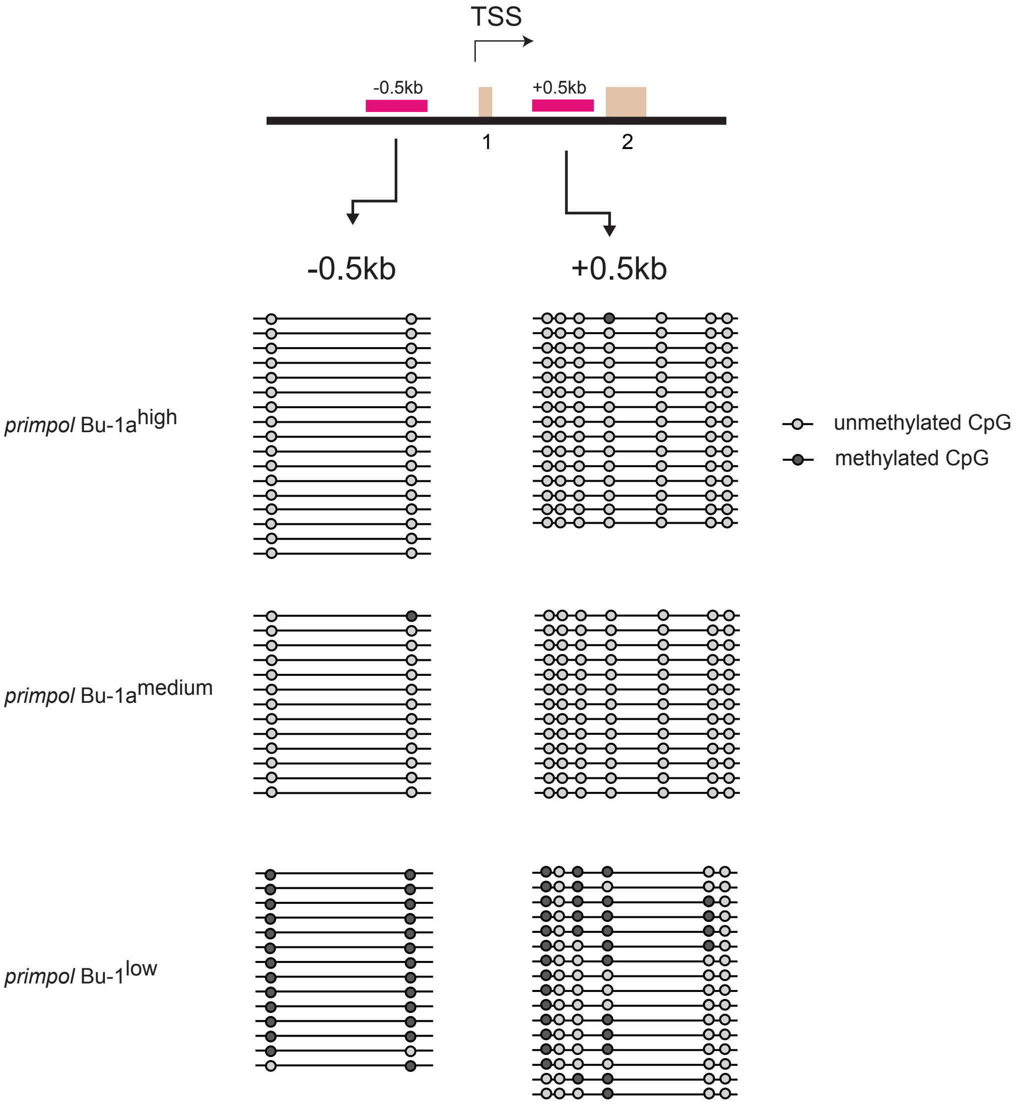

Figure S2. DNA methylation of the *BU-1* promoter determined by bisulphite sequencing. Related to Figure 1.

Bisulphite sequencing results for primers spanning regions located approximately 0.5 kb up and downstream of the TSS of *BU-1*. CpG sites are indicated as circles. Open circles = unmethylated; filled circles = methylated.

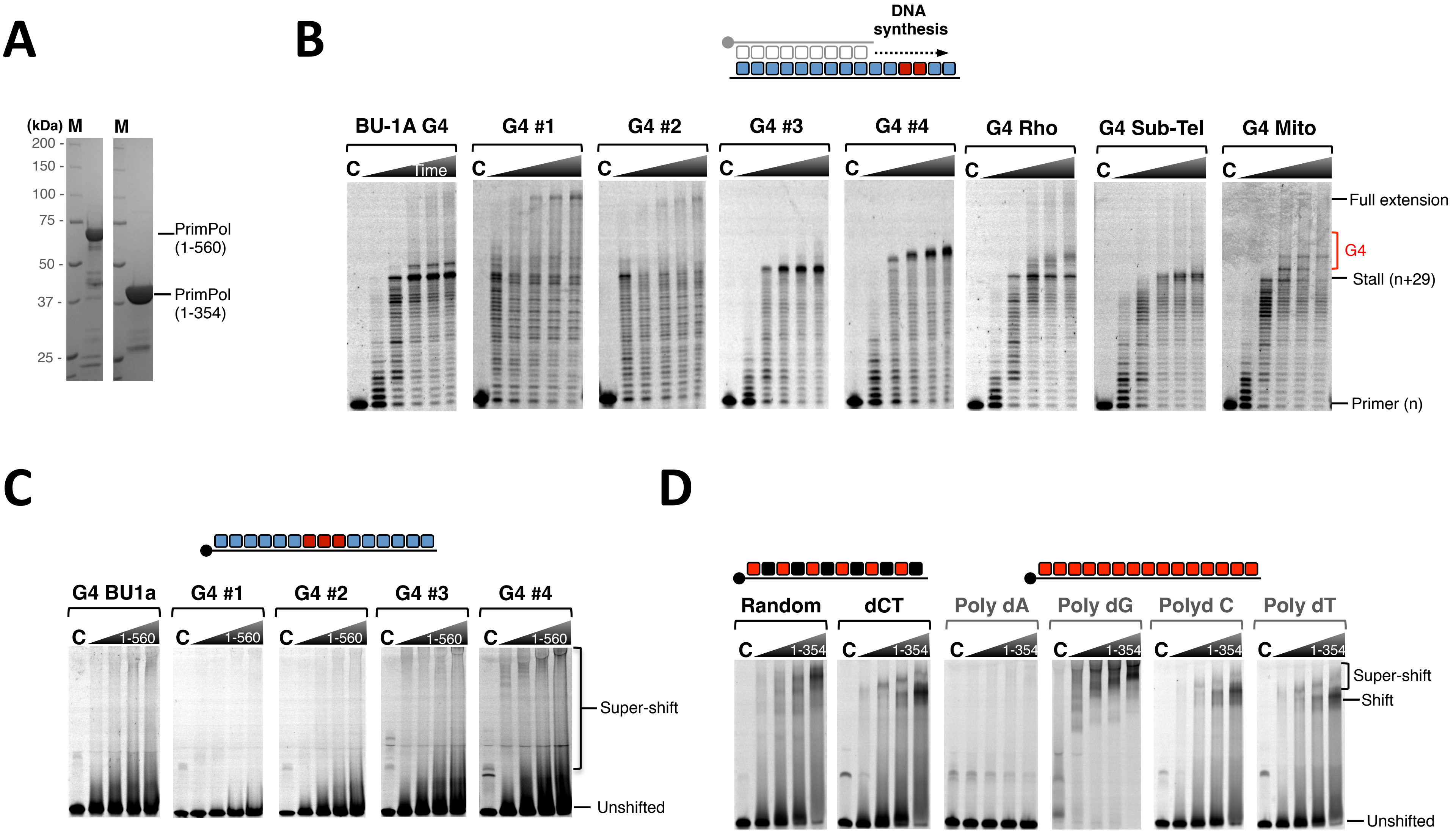

A
(kDa)
M
M
200 -
150 -
100 -
75 -
50 -
37 -
25 -
PrimPol (1-560)
PrimPol (1-354)
B
DNA synthesis
BU-1A G4
G4 #1
G4 #2
G4 #3
G4 #4
G4 Rho
G4 Sub-Tel
G4 Mito
C
Time
Full extension
G4
Stall (n+29)
Primer (n)
C
G4 BU1a
G4 #1
G4 #2
G4 #3
G4 #4
1-560
Super-shift
Unshifted
D
Random
dCT
Poly dA
Poly dG
Polyd C
Poly dT
1-354
Super-shift
Shift
Unshifted

Figure S3. Preparation of recombinant human PrimPol proteins, primer extensions and electrophoretic mobility shift assays. Related to Figure 3.

A. SDS PAGE analysis of human recombinant PrimPol proteins. Histidine-tagged full-length human PrimPol (1-560) was purified using Ni-NTA affinity purification, heparin, and size-exclusion chromatography. Glutathione S-transferase (GST) tagged truncation of human PrimPol (1-354) was purified using Glutathione affinity chromatography. The GST tag was cleaved off by incubation with prescission protease over night at 4°C. Subsequently, the tagless truncation of PrimPol (1-354) was purified by heparin and size-exclusion chromatography.

B. Histidine-tagged full length PrimPol (1-560) at 100 nM was incubated with 20 nM G4 quadruplex containing substrates and 200 μM dNTPs for increasing times (1, 2, 5, 10, 20 minutes) at 37°C. A no enzyme control ("C") was performed as a single 20 minute time point at 37°C in the absence of PrimPol.

C. Increasing concentrations (0.7, 2.5, 5, 10 μM) of $\text{PrimPol}_{1\text{-}560}$ were incubated with 100 nM fluorescently labelled ssDNA probes. A no enzyme control ("C") was also performed to monitor mobility of ssDNA probes. The G4 quadruplex structures are marked as red boxes.

D. Increasing concentrations (0.7, 2.5, 5, 10 μM) of $\text{PrimPol}_{1\text{-}354}$ were incubated with 100 nM fluorescently labelled ssDNA probes. A no enzyme control ("C") was also performed to monitor the mobility of ssDNA probes. The homopolymeric nucleotide runs are marked as red boxes and mixed sequences as red and black boxes.

Schiavone, Jozwiakowski et al. Figure S4

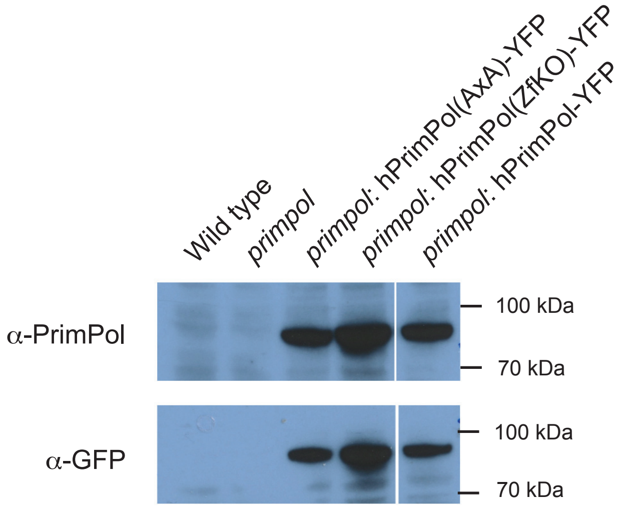

Figure S4. Western blot of YFP-tagged human PrimPol and mutant derivatives in *primpol* DT40 cells. Related to Figure 4.

Whole cell lysates blotted with anti-human PrimPol and anti-GFP. Note the anti-human PrimPol antibody does not cross-react against the chicken protein.

Supplemental Experimental Procedures

Primers for ChIP qPCR
(*c.* -0.5kb and +0.5kb from the *BU-1* TSS)

ChIP -0.5 F	TAGCTCCAAGGTGTGGGACTTT
ChIP -0.5 R	CCCCATACTGGACAGACTGAATA
ChIP +0.5 F	GGCAGCTCAGCAAAGTTTCC
ChIP +0.5 R	GACCACAGCCGTGGAACAGTTA

Primers for methylation analysis
(*c.* -0.5kb and +0.5kb from the *BU-1* TSS)

Methyl -0.5 F	GTTTCTT**GAGCTC**TTTGGTAAGTGATAGTTATTGGTATTGTA
Methyl -0.5 R	GTTTCTT**GCGGCCGC**ATTAACTAAACTCAAACTAAACCAACAC
Methyl +0.5 F	GTTTCTT**GAGCTC**GAATATAAGGTTTTGGTATGTAGAATGT
Methyl +0.5 R	GTTTCTT**GCGGCCGC**CTCCCTAATCACTAAAATTATATACAAAA

Colour code: (Pig tail) SacI/NotI

PCR primers for hPrimPol

Amplicon	Forward Primer (5'→3')	Reverse Primer (5'→3')
hPrimPol$_{1-354}$	GTTTCTTGGATCCATGAATA GAAAATGGGAAGCAAAAC	CTTTGTTGCGGCCGCTTACTCTTGTAATACTTCTATAATT AGTTCATCAGGAATTTC

Synthetic primer-template substrates

Figure (Substrate)	Primer (5'→3')	Template (5'→3')
S3B (G4 BU1a)	TGTCGTCTGTTCGGTC GTTC	ACCGCGAACTTGAATTCTAGGGCTGGGTGGGTGCTGT CAAGGGCTGGGCAATGCACAACATATGGCTTTCGAAG ACCGAACGACCGAACAGACGACA
S3B (G4#1)	TGTCGTCTGTTCGGTC GTTC	ACCGCGAACTTGAATTCTATTGGTTTTGGTTTTGGTTTT GGTCAATGCACAACATATGGCTTTCGAAGACCGAACGA CCGAACAGACGACA
S3B (G4#2)	TGTCGTCTGTTCGGTC GTTC	ACCGCGAACTTGAATTCTATGGGTTTGGGTTTGGGTTT GGGTCAATGCACAACATATGGCTTTCGAAGACCGAAC GACCGAACAGACGACA
S3B (G4#3)	TGTCGTCTGTTCGGTC GTTC	ACCGCGAACTTGAATTCTATGGGGTTGGGGTTGGGGT TGGGGCAATGCACAACATATGGCTTTCGAAGACCGAA CGACCGAACAGACGACA
S3B (G4#4)	TGTCGTCTGTTCGGTC GTTC	ACCGCGAACTTGAATTCTATTTTGGGTGGGTGGGTGG GTTTTCAATGCACAACATATGGCTTTCGAAGACCGAAC GACCGAACAGACGACA
S3B (G4 Rho)	TGTCGTCTGTTCGGTC GTTC	ACCGCGAACTTGAATTCTAGGGAGTAAAAGGGAGCG GGTGCTGGGGCAATGCACAACATATGGCTTTCGAAGA CCGAACGACCGAACAGACGACA

S3B (G4 Sub-Tel)	TGTCGTCTGTTCGGTC GTTC	ACCGCGAACTTGAATTCTATTAGGGTTAGGGTTAGGGTTAGGGCAATGCACAACATATGGCTTTCGAAGACCGAACGACCGAACAGACGACA
S3B (G4 Mito)	TGTCGTCTGTTCGGTC GTTC	ACCGCGAACTTGAATTCTAGGGTGGGATGGGCGGGGGCAATGCACAACATATGGCTTTCGAAGACCGAACGACCGAACAGACGACA

Synthetic ssDNA probes

Figure (Substrate)	ssDNA probe (5'→3')
F3A (G4 BU1a)	CTTGAATTCTAGGGCTGGGTGGGTGCTGTCAAGGGCTGGGCACATATGTTC
F3A (G4#1)	CTTGAATTCTATTGGTTTTGGTTTTGGTTTTGGTCACATATGTTC
F3A (G4#2)	CTTGAATTCTATGGGTTTGGGTTTGGGTTTGGGTCACATATGTTC
F3A (G4#3)	CTTGAATTCTATGGGGTTGGGGTTGGGGTTGGGGCACATATGTTC
F3A (G4#4)	CTTGAATTCTATTTTGGGTGGGTGGGTGGGTTTTCACATATGTTC
F3B (Random)	CGCGCAGGGCGCACAACAGCCTTGAAGACCGAACGACCGAACAGACGACA
F3B (dCT)	CTCTCTCTCTCTCTCTCTCTCTCTCTCTCTCTCTCTCTCTCTCTCTCTCT
F3B (Poly dA)	AAAAAAAAAAAAAAAAAAAAAAAAAAAAAAAAAAAAAAAAAAAAAAAAAA
F3B (Poly dC)	CCCCCCCCCCCCCCCCCCCCCCCCCCCCCCCCCCCCCCCCCCCCCCCCCC
F3B (Poly dT)	TTTTTTTTTTTTTTTTTTTTTTTTTTTTTTTTTTTTTTTTTTTTTTTTTT
F3B (Poly dG)	GGGGGGGGGGGGGGGGGGGGGGGGGGGGGGGGGGGGGGGGGGGGGGGGGG
F3C (dA12)	CTTGAATTCTT**AAAAAAAAAAAA**TCGTAGCAGTACACATATGTTC
F3C (dC12)	CTTGAATTCTA**CCCCCCCCCCCC**ACGTAGCAGTACACATATGTTC
F3C (dT12)	CTTGAATTCTA**TTTTTTTTTTTT**ACGTAGCAGTACACATATGTTC
F3C (dG12)	CTTGAATTCTA**GGGGGGGGGGGG**ACGTAGCAGTACACATATGTTC
F3C (dG9)	CTTGAATTCTAAC**GGGGGGGGG**TACGTAGCAGTACACATATGTTC
F3C (dG6)	CTTGAATTCTAACA**GGGGGG**TATACGTAGCAGTACACATATGTTC
S3C (G4 BU1a)	CTTGAATTCTAGGGCTGGGTGGGTGCTGTCAAGGGCTGGGCACATATGTTC
S3C (G4#1)	CTTGAATTCTATTGGTTTTGGTTTTGGTTTTGGTCACATATGTTC
S3C (G4#2)	CTTGAATTCTATGGGTTTGGGTTTGGGTTTGGGTCACATATGTTC
S3C (G4#3)	CTTGAATTCTATGGGGTTGGGGTTGGGGTTGGGGCACATATGTTC
S3C (G4#4)	CTTGAATTCTATTTTGGGTGGGTGGGTGGGTTTTCACATATGTTC

S3D (Random)	CGCGCAGGGCGCACAACAGCCTTGAAGACCGAACGACCGAACAGACGACA
S3D (dCT)	CTCTCTCTCTCTCTCTCTCTCTCTCTCTCTCTCTCTCTCTCTCTCTCTCT
S3D (Poly dA)	AAAAAAAAAAAAAAAAAAAAAAAAAAAAAAAAAAAAAAAAAAAAAAAAAA
S3D (Poly dG)	GGGGGGGGGGGGGGGGGGGGGGGGGGGGGGGGGGGGGGGGGGGGGGGGGG
S3D (Poly dC)	CCCCCCCCCCCCCCCCCCCCCCCCCCCCCCCCCCCCCCCCCCCCCCCCCC
S3D (Poly dT)	TTTTTTTTTTTTTTTTTTTTTTTTTTTTTTTTTTTTTTTTTTTTTTTTTT

Primers for BU-1 amplification and G4 locus sequencing

(*Fragments A, B and C*)

Fragm_A_F	GTTTCTTGCGGCCGCTCGATCCTTATGGGTCCCTTCTA
Fragm_A_R	GTTTCTTCTCGAGGCTCCTAAAAGCTCAAGAGGTCT
Fragm_B_F	GTTTCTTGCGGCCGCCTCTGTAGCCAGATCGTCTTCTC
Fragm_B_R	GTTTCTTCTCGAGTTCATACCAACTCTACACCAGCC
Fragm_C_F	GTTTCTTGCGGCCGCGACATTCACCCATAACCCTGACT
Fragm_C_R	GTTTCTTCTCGAGGAGATCTCTGGTGGTTCCTTGTT
G4seq	GTAGGAAGTAGGCTGGTTCCTTGC

Pigtail NotI/XhoI

Primase assay substrates

**Figure (Substrate)**	**Primer (5'→3')**	**Template (5'→3')**
F3D (G4, left)	GATGCATCTATGTddG	GTCTTCTATCTCGTCTATATTCTATTGTCTCTATGGGTG GGTGGGTGGGTCTCACATAGATGCATC
F3D (Control, left)	GATGCATCTATGTddG	GTCTTCTATCTCGTCTATATTCTATTGTCTCTATGAATA CCTTCATCAGTCTCACATAGATGCATC
F3D (G4, right)	N/A	GTCTTCTATCTCGTCTATATTCTATTGTCTCTATGGGTG GGTGGGTGGGTCTCA
F3D (Control, right)	N/A	GTCTTCTATCTCGTCTATATTCTATTGTCTCTATGAATA CCTTCATCAGTCTCACATAGATGCATC
