# Supplementary material for: PrimPol Is Required for Replicative Tolerance of G Quadruplexes in Vertebrate Cells
Source: Mol Cell. 2016 Jan 7;61(1):161–9. doi: 10.1016/j.molcel.2015.10.038 (PMC4712188; doi:10.1016/j.molcel.2015.10.038)
Supplement: Document S2. Article plus Supplemental Information [file mmc2.pdf]

Short Article

Molecular Cell

PrimPol Is Required for Replicative Tolerance of G Quadruplexes in Vertebrate Cells

Graphical Abstract

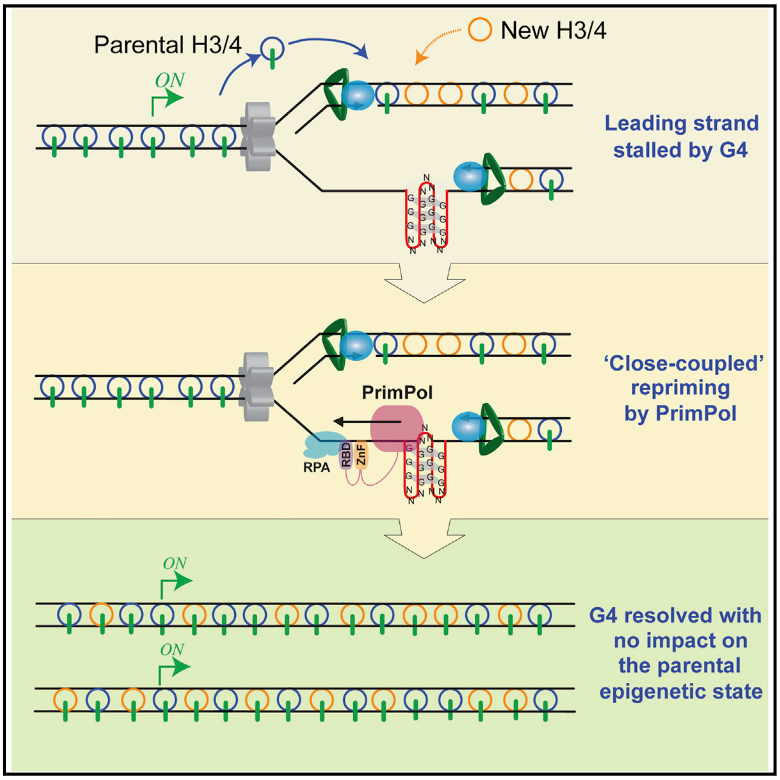

Authors

Davide Schiavone, Stanislaw K. Jozwiakowski, Marina Romanello, ..., Laura J. Bailey, Julian E. Sale, Aidan J. Doherty

Correspondence

jes@mrc-lmb.cam.ac.uk (J.E.S.), ajd21@sussex.ac.uk (A.J.D.)

In Brief

Schiavone, Jozwiakowski et al. demonstrate that the recently described primase-polymerase in vertebrates, PrimPol, plays an important role in replicating G quadruplex (G4) structures by binding them and repriming DNA-leading strand synthesis close by. This maintains processive replication and prevents disruption of histone recycling, ensuring epigenetic stability around G4s.

Highlights

- G4s block replication in cells lacking PrimPol causing local epigenetic instability
- PrimPol binds G4s but cannot directly replicate them
- PrimPol reprimes DNA synthesis closely coupled to G4s
- Repriming preserves epigenetic stability in proximity G4 sequences

Schiavone et al., 2016, Molecular Cell *61*, 161–169
January 7, 2016 ©2016 The Authors
http://dx.doi.org/10.1016/j.molcel.2015.10.038

CrossMark

CellPress

PrimPol Is Required for Replicative Tolerance of G Quadruplexes in Vertebrate Cells

Davide Schiavone,[1,3] Stanislaw K. Jozwiakowski,[2,3] Marina Romanello,[1] Guillaume Guilbaud,[1] Thomas A. Guilliam,[2] Laura J. Bailey,[2] Julian E. Sale,[1,*] and Aidan J. Doherty[2,*]
[1]MRC Laboratory of Molecular Biology, Francis Crick Avenue, Cambridge, CB2 0QH, UK
[2]Genome Damage and Stability Centre, School of Life Sciences, University of Sussex, Brighton, BN1 9RQ, UK
[3]Co-first author
*Correspondence: jes@mrc-lmb.cam.ac.uk (J.E.S.), ajd21@sussex.ac.uk (A.J.D.)
http://dx.doi.org/10.1016/j.molcel.2015.10.038
This is an open access article under the CC BY license (http://creativecommons.org/licenses/by/4.0/).

SUMMARY

G quadruplexes (G4s) can present potent blocks to DNA replication. Accurate and timely replication of G4s in vertebrates requires multiple specialized DNA helicases and polymerases to prevent genetic and epigenetic instability. Here we report that PrimPol, a recently described primase-polymerase (PrimPol), plays a crucial role in the bypass of leading strand G4 structures. While PrimPol is unable to directly replicate G4s, it can bind and reprime downstream of these structures. Disruption of either the catalytic activity or zinc-finger of PrimPol results in extreme G4-dependent epigenetic instability at the *BU-1* locus in avian DT40 cells, indicative of extensive uncoupling of the replicative helicase and polymerase. Together, these observations implicate PrimPol in promoting restart of DNA synthesis downstream of, but closely coupled to, G4 replication impediments.

INTRODUCTION

G4s are DNA secondary structures formed by the stacking of quartets of Hoogsteen-bonded guanine bases. Extensively characterized in vitro, steadily accumulating evidence supports their formation in vivo (Murat and Balasubramanian, 2014) with a contribution to cellular function, including regulation of transcription and initiation of DNA replication (Maizels and Gray, 2013). However, they can also pose significant impediments to DNA replication resulting in both genetic and epigenetic instability (Cheung et al., 2002; Ribeyre et al., 2009; Sarkies et al., 2010). Interference with replication is likely to be a common problem as the human genome is estimated to contain between 350,000 and 700,000 potential G4-forming sites (Maizels and Gray, 2013). This abundance of G4 motifs might explain why vertebrate cells have evolved numerous mechanisms to ensure their efficient and accurate replication. To date, there is evidence that several specialized DNA helicases, including FANCJ, PIF1, WRN, and BLM, and polymerases, Polη, Polκ, and REV1, are involved in replicating G4 structures (León-Ortiz et al., 2014; Wickramasinghe et al., 2015). However, this is almost certainly an incomplete list of the factors required to ensure efficient replication of these structures in vivo.

Recently, a DNA polymerase called Primase-Polymerase (PrimPol) has been implicated in eukaryotic DNA damage tolerance (Bianchi et al., 2013; Rudd et al., 2013; Wan et al., 2013; García-Gómez et al., 2013; Mourón et al., 2013). PrimPol is required for the bypass of photo and oxidative lesions during both nuclear and mitochondrial replication in eukaryotes, contributing both re-priming and translesion synthesis activities. It is required for maintaining replication fork progression on UV-damaged templates, as assessed in isolated DNA fibers, suggesting that it acts at, or close to, the replication fork (Rudd et al., 2014). PrimPol is also required during unperturbed replication, with significant replication slowing observed in PrimPol knockout cells (hereafter *primpol* cells) followed by the appearance of chromosomal breaks. Indeed, it is an essential gene in trypanosomes (Rudd et al., 2013). Since PrimPol has low processivity and is highly biased toward insertion-deletion (indel) errors (Guilliam et al., 2015; Keen et al., 2014), its use needs to be tightly controlled. However, unlike the Y-family polymerases, it is not regulated by PCNA (Guilliam et al., 2015). Rather, PrimPol interacts with the major single-strand binding proteins (SSBs), RPA and mtSSB (Guilliam et al., 2015; Wan et al., 2013), both SSBs significantly limiting both its primase and polymerase activities, thereby potentially limiting its capacity for mutagenesis at the replication fork (Guilliam et al., 2015).

Although the role of PrimPol in damage tolerance has been established, its intrinsic capacity to bypass distorting lesions (e.g., 6-4 photoproducts) suggests that it might have additional potential in assisting the bypass of DNA secondary structures formed during replication, such as G4s.

RESULTS AND DISCUSSION

To examine the possibility that PrimPol contributes to G4 replication in vivo, we took advantage of a recently described assay that monitors G4 replication through the stochastic downregulation of transcription of the *BU-1A* locus in chicken DT40 cells (Schiavone et al., 2014; Sarkies et al., 2012). The six exons of the *BU-1* locus encode a surface glycoprotein whose expression can be monitored with specific antibodies. The locus contains a G4

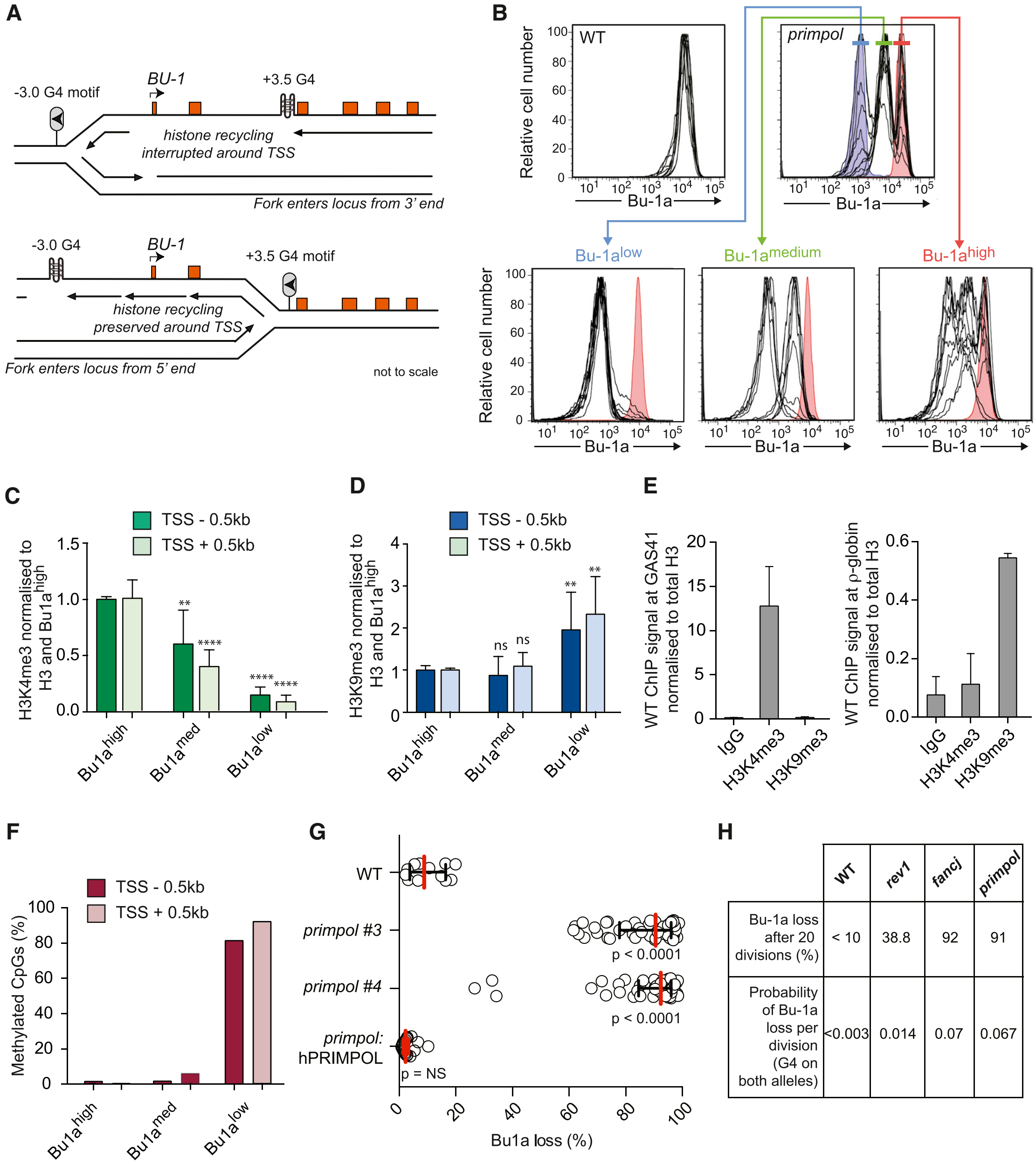

Figure 1. PrimPol Is Required to Maintain Epigenetic Stability of the *BU-1* Locus of DT40 Cells

(A) The *BU-1* locus. The leading strand of a replication fork entering the locus from the right stalls at the +3.5 G4 (lollipop). This leads to the formation of a putative post-replicative gap and loss of key histone modifications in a zone of interrupted histone recycling extending up to 4.5 kb from the +3.5 G4 motif (Schiavone et al., 2014).

(B) Instability of Bu-1a expression in *primpol* cells. Top: FACS plots of wild-type and *primpol* DT40 cells stained with anti-Bu-1a conjugated to phycoerythrin. Each line represents the Bu-1a expression profile of an individual clonal population. Bu-1a^{high} and unstained controls are shown in red and blue, respectively, in the *primpol* panel. Subclones expanded from each population of *primpol* cells (Bu-1a^{high}, red; Bu-1a^{medium}, green; Bu-1a^{low}, blue) are shown in the bottom panel. For reference, a Bu-1a^{high} wild-type profile is shown in red.

(C) H3K4me3 upstream (dark green) and downstream (light green) of the TSS of *BU1A* in the three populations of *primpol* cells. The specific ChIP signal was normalized first to H3 and then to the level in Bu-1a^{high} cells. p values relative to Bu-1a^{high} **$p < 0.01$; ****$p < 0.0001$ (Welch's t test).

(D) H3K9me3 upstream (dark blue) and downstream (light blue) of the TSS of *BU-1A* in the three populations of *primpol* cells. The specific ChIP signal was normalized first to H3 and then to the level in Bu-1a^{high} cells. Statistics are as in (C).

(E) ChIP controls. Enrichment of H3K4me3 at the promoter of the transcriptionally active *GAS41* locus. Enrichment of H3K9me3 at the repressed ρ-globin locus. The ChIP signals are normalized to total H3.

(legend continued on next page)

motif within the second intron, 3.5 kb downstream of the transcription start site (TSS) (Figure 1A). In cells lacking enzymes needed for effective G4 replication, such as the REV1 polymerase or FANCJ helicase, replication stalling at the +3.5 G4 results in stochastic loss of wild-type levels of Bu-1a expression as cells divide (Sarkies et al., 2012; Schiavone et al., 2014). We previously provided evidence that this transcriptional instability results from localized loss of coupling between DNA synthesis and histone recycling and failure to accurately propagate the parental pattern of histone modifications. In the *BU-1A* locus, this results in loss of H3K4me3 and H3K9ac around the TSS and hence loss of high level Bu-1a expression (Schiavone et al., 2014). The rate at which Bu-1a loss variants are generated can be measured by fluctuation analysis, allowing a per division probability of losing Bu-1a^{high} expression to be calculated (Schiavone et al., 2014).

To establish whether loss of PrimPol gives rise to transcriptional instability of *BU-1A*, we monitored surface Bu-1a expression in *primpol* cells (Bianchi et al., 2013). As expected, wild-type DT40 cells are uniformly Bu-1a^{high} (Figure 1B). Strikingly, however, PrimPol-deficient cells exhibited three discrete levels of Bu-1a expression (Figure 1B), Bu-1a^{high}, Bu-1a^{medium}, and Bu-1a^{low}, the last of which has essentially lost Bu-1a expression. Through sorting these populations, we found that Bu-1a^{medium} and Bu-1a^{low} cells arise spontaneously, sequentially, and irreversibly from Bu-1a^{high} populations (Figure 1B). Sequencing around the +3.5 G4 and restriction mapping of the whole *BU-1* locus in Bu-1a^{low} cells revealed no evidence of mutation or gross genetic instability (Figure S1). However, chromatin immunoprecipitation from each population revealed that the Bu-1a^{medium} population exhibits reduction in the "active" mark H3K4me3 at the *BU-1* promoter, but no increase in "repressive" H3K9me3 or DNA methylation (Figures 1C–1F and S2). The Bu-1a^{low} population had not only lost H3K4me3 but also exhibited significant enrichment of H3K9me3 and DNA methylation (Figures 1C–1F and S2). Thus, lack of PrimPol results in spontaneous and stochastic silencing of the *BU-1A* locus, first with loss of parental H3K4me3 followed by heterochromatinization.

To estimate the frequency with which Bu-1a^{high} expression is lost in *primpol* cells, we performed a fluctuation analysis (Schiavone et al., 2014). Bu-1a^{high} *primpol* cells were isolated and expanded from *c.* two to *c.* 10^6 cells, approximating to an average of 20 divisions, after which the proportion of cells that had lost wild-type levels of Bu-1a expression was monitored by flow cytometry. Compared with wild-type cells, the two independent *primpol* mutants tested exhibited extremely high-frequency generation of Bu-1a loss variants (Figure 1G). This instability of *BU-1A* expression was fully complemented by expression of human PrimPol (Figure 1G). Using our previously described Monte Carlo simulation (Schiavone et al., 2014), we estimated that the rate of loss of full *BU-1A* expression in *primpol* equated to a per division probability of 0.067. This loss was nearly 5-fold higher than observed in the *rev1* mutant but comparable to the instability observed in *fancj* cells grown under the same conditions (Figure 1H).

To test whether the instability of Bu-1a expression was due to the +3.5 G4, we deleted the motif on both alleles of *BU-1* by homologous gene targeting. This resulted in complete stabilization of expression of Bu-1a (Figure 2A). Reintroduction of the +3.5 G4 motif back into its original position in the *BU-1A* allele resulted once again in unstable expression of the gene (Figure 2B), confirming that the structure is necessary and sufficient to cause the epigenetic instability of the locus. To assess whether the instability of the *BU-1* locus was dependent on the orientation of the G4 motif, we reintroduced an inverted version of the +3.5 G4. In this orientation, the G4 motif did not induce instability (Figure 2C). This is consistent with arrest of lagging strand synthesis in the body of the gene not resulting in gaps of sufficient length to affect histone propagation around the promoter, as we have previously reported in REV1-deficient cells (Schiavone et al., 2014).

We next asked whether PrimPol is required at particular types of G4. The *BU-1* +3.5 G4 motif forms hybrid parallel/anti-parallel structures in vitro but has a long (9 base pair) non-G loop and intermediate T_m of 65.5°C (Schiavone et al., 2014). We therefore also assessed the impact of two model G4 motifs with very different in vitro properties. G4#1 (Schiavone et al., 2014) (5′-TTGGTTTTGGTTTTGGTTTTGGT-3′) forms a predominantly anti-parallel structure in K^+ ions but has a low in vitro melting temperature of under 30°C (Figures 2D–2F). In contrast, G4#4 (Schiavone et al., 2014) (5′-TTTTGGGTGGGTGGGTGGGTTTT-3′) forms a very thermally stable (~95°C), predominantly parallel structure. In REV1-deficient cells, we previously reported that Bu-1a instability correlated with non-G loop length but surprisingly not with in vitro T_m (Schiavone et al., 2014). Strikingly, in the *primpol* mutant, this relationship was reversed (Figures 2D–2F) with the greatest instability being observed with G4#4, which also has the highest in vitro Tm.

To understand the basis for these in vivo observations, we next examined the biochemical activity of PrimPol at G4 structures. Replicative polymerases are unable to synthesize effectively through G4s, which thus present a profound replication block in vitro (Woodford et al., 1994). We therefore presented purified PrimPol (Figure S3A) with a variety of G4 secondary structures (the *BU-1*, or model G4s). PrimPol had some limited capacity to synthesize through G4s formed in the presence of sodium ions (data not shown), which result in structures that

(F) DNA methylation analysis. Percentage of methylated CpG sites in a region −0.5 kb from the TSS (dark red) and +0.5 kb from the TSS (light red) in the three Bu-1a expression states. The position of the primers used and a schematic of the raw data are presented in Figure S2.

(G) Fluctuation analysis for Bu-1a loss in wild-type cells, two independent *primpol* clones, and one *primpol* clone complemented by expression of human PrimPol. Each circle represents the percentage of cells in an individual clone expanded for 3 weeks that have lost Bu-1a^{high} expression (i.e., are Bu-1a^{med} or Bu-1a^{low}). Bars and whiskers represent median and interquartile range, respectively. p values calculated by Fisher's exact test using bin sizes of 20%.

(H) Per-division probability of loss of Bu-1a^{high} expression in *primpol* compared with wild-type, *rev1*, and *fancj* mutants. Probability of a cell loosing Bu-1a^{high} expression was calculated using our previously published Monte Carlo simulation, assuming the presence of the +3.5 G4 motif on both alleles to account for the transvection-like effect between the alleles (Schiavone et al., 2014). The intrinsic noise of this FACS-based assay prevents accurate determination per division probability when loss populations are <10%. See also Figures S1 and S2.

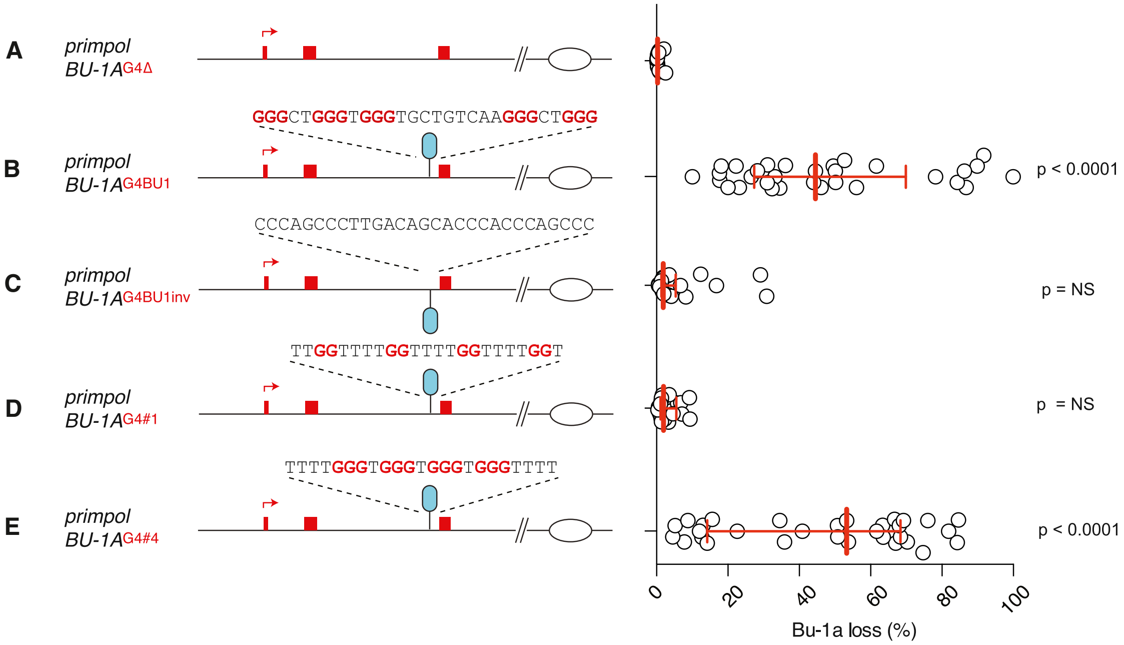

F

	rev1			*primpol*		
	BU-1A G4	G4 #1	G4 #4	*BU-1A* G4	G4 #1	G4 #4
In vitro Tm (°C)	65.5	27.6	> 95	65.5	27.6	> 95
Max. non G loop	9	4	1	9	4	1
Predominant topoogy	hybrid	anti-parallel	parallel	hybrid	anti-parallel	parallel
Bu-1a loss after 20 divisions (%)	33.1	22.1	< 10*	44.5	< 10*	53.3
Probability of Bu-1a loss per division (G4 on one allele)	0.021	0.012	< 0.003	0.03	< 0.003	0.039

Figure 2. Determinants of Bu-1a Instability in *primpol* Cells

Fluctuation analyses for Bu-1a loss in clones of the indicated *BU-1A* genotype. All experiments were carried out in cells in which the +3.5 G4 motif had been deleted on both the *BU-1A* and *BU-1B* alleles to avoid any influence of the previously described transvection-like effect between the two alleles (Schiavone et al., 2014).

(A) Bu-1a loss in *primpol* cells in which the +3.5 G4 motif has been deleted on both the *BU-1A* and *BU-1B* alleles.

(B) Bu-1a loss in *primpol* cells in which the +3.5 G4 motif has been reintroduced into the *BU-1A* allele. Note the level of instability is approximately 50% of that seen in *primpol* cells (Figure 1G) due to a transvection-like effect between the two alleles in which loss of expression in one leads to loss of expression in the other (Schiavone et al., 2014).

(C) Bu-1a loss following reintroduction of the +3.5 G4 motif inverted so that the G-rich strand is now on the lagging strand template for a fork entering the locus from the right.

(D) Bu-1a loss following replacement of the +3.5 G4 motif with a thermodynamically weak G4 motif, which in vitro forms a predominantly anti-parallel structure with a Tm of 27°C (Schiavone et al., 2014).

(E) Bu-1a loss following replacement of the +3.5 G4 motif with a thermodynamically strong G4 motif, which in vitro forms a predominantly parallel structure with a Tm of >95°C (Schiavone et al., 2014).

(F) Per-division probability of loss of Bu-1a^{high} expression with different G4s. The indicated G4s were knocked into the *BU-1A* allele of wild-type and *primpol* *BU-1A*$^{\Delta G4}$/*BU-1B*$^{\Delta G4}$ cells in the position of the +3.5 G4. Biophysical data is taken from (Schiavone et al., 2014). The Monte Carlo simulation was modified to account for a G4 being present only on the *BU-1A* allele.

are less stable than those formed in K$^+$ (Hardin et al., 1992). However, it was incapable of synthesizing through template strands containing G4 structures formed in the presence of 50 mM potassium ions, exhibiting significant stalling at these "roadblocks" (Figure S3B). We also repeated the assays in the presence of auxiliary factors (RPA and PCNA). However, no significant increase in G4 bypass was observed (data not shown). Together, these data establish that human PrimPol, on its own or in the presence of additional replication factors, stalls at G4s and is unable to effectively perform template-dependent synthesis through these secondary structures in vitro.

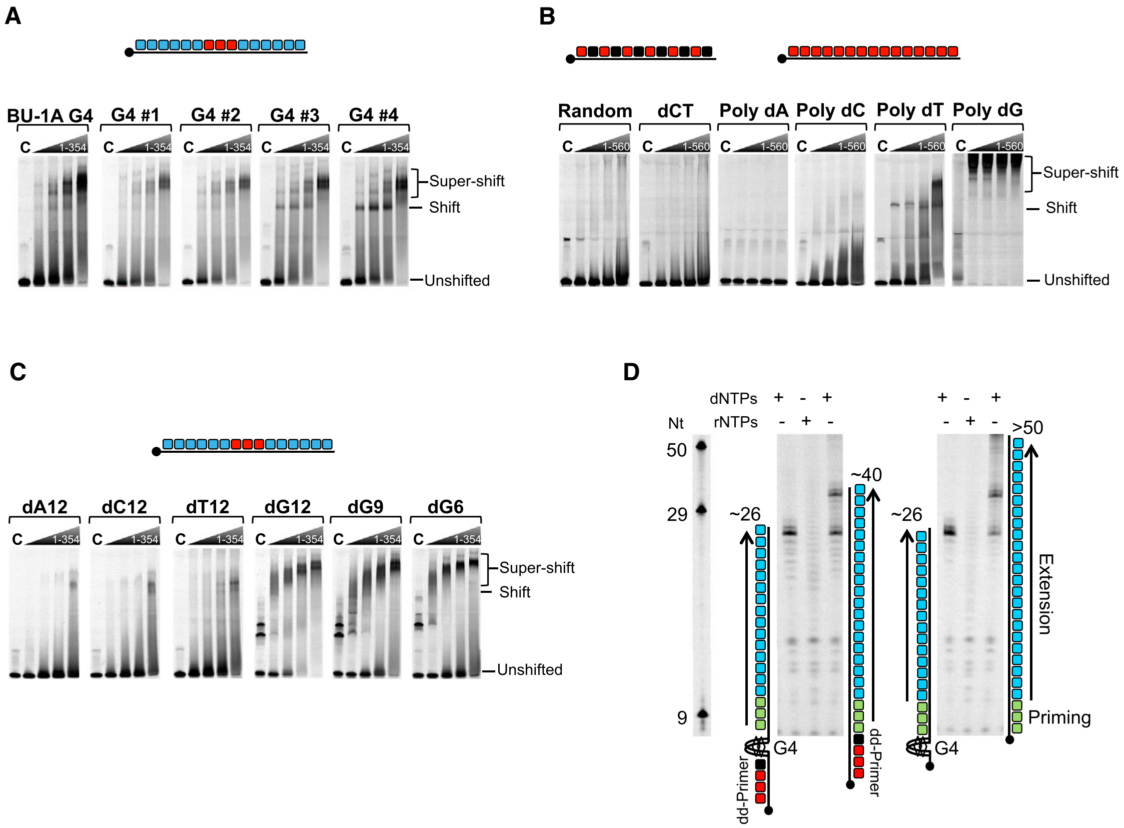

Figure 3. PrimPol Binds to Homopolymeric dG and G4 DNA Sequences and Can Catalyze Close-Coupled Repriming Downstream of a G4 Structure

(A) Panels show the EMSA binding profiles for PrimPol$_{1\text{-}354}$ incubated with a number G4 structures formed from ss DNAs containing flanking mixed sequences with a central G4 sequence repeat composed of: Bu-1A or G4 variants (G4 #1 to G4 #4) with decreasing loop sizes from 4 to 1, respectively. Increasing concentrations of PrimPol $_{1\text{-}354}$ (0.7, 2.5, 5, and 10 μM) were incubated with 100 nM of fluorescently labeled ssDNA probes. No protein control ("C") was also performed to monitor mobility of "free" ssDNA probes.

(B) Panels show the EMSA binding patterns for increasing concentrations full-length PrimPol (0.7, 2.5, 5, and 10 μM) incubated with a variety of 50-mer ss DNAs composed of: mixed sequence (random), alternate dCT, or homopolymeric (dA, dC, dT, or dG) DNA substrates.

(C) Increasing concentrations of PrimPol$_{1\text{-}354}$ (0.7, 2.5, 5, and 10 μM) incubated with a variety of ss DNAs containing flanking mixed sequences with a central short homopolymeric repeat composed of 12nt homopolymeric sequences (dA, dC, dT, or dG) or poly dG of varying lengths (6, 9, or 12mers).

(D) PrimPol (2 μM) was incubated for 30 min at 37°C with dNTPs or rNTPs (250 μM), FAM-dNTPs (dATP, dCTP, dUTP) (2.5 μM), and mixed sequence G4-containing or control templates (1 μM) (as shown in the schematic). Identical reactions were also performed with rNTPs (250 μM) instead of dNTPs on the G4 containing templates only (middle lanes). Templates were either annealed to primers containing a 3′ dideoxynucleotide (shown in red) upstream of the G4 structure or contained only a short sequence (5 nt) before the structure. Priming and extension are represented as green and blue, respectively. The length of products extended to the end of the template by PrimPol allows analysis of the priming location; identically sized extension products on both G4-containing templates reveals close-coupled re-priming downstream of the G4 structure in each case. Oligonucleotide nucleotide (Nt) length markers are shown in the left panel. See also Figure S3.

Although full-length PrimPol has limited binding affinity for DNA (Keen et al., 2014), the catalytic domain (PrimPol$_{1\text{-}354}$) binds proficiently to both ss and ds DNA. To investigate the affinity of PrimPol$_{1\text{-}354}$ for specific G4 structures, we performed electrophoretic mobility shift assays (EMSAs) with a +3.5 *BU-1A* G4 DNA substrate formed in the presence of 50 mM potassium ions. Specific shifted species were observed upon addition of increasing concentrations of PrimPol$_{1\text{-}354}$ to the *BU-1A* G4 structure (Figure 3A), indicating that this enzyme can also bind to folded G4 structures. To investigate whether G4 loop size plays a role in PrimPol binding, we repeated the EMSAs with G4 substrates possessing loops of decreasing length between 4 and 1 (G4#1–4). PrimPol$_{1\text{-}354}$ formed shifted intermediates with G4#1 and G4#2 (Figures 3A and S3C), which contain non-G loops of 4 and 3, respectively. However, more prominent species were observed with G4#3 and, particularly, with G4#4 that contain loops of 2 and 1, respectively, and that form more thermodynamically stable structures. Together, these findings demonstrate that PrimPol can bind to template DNA containing G4 structures, with greater affinity for quadruplexes with short loops and higher thermal stability.

Homopolymeric dexoyguanosine can form inter- and intramolecular G4 structures in solution (Sengar et al., 2014). To investigate whether PrimPol also exhibits binding preferences

for homopolymeric DNA, we performed EMSAs on a set of single-stranded homopolymeric DNA sequences (50-mer poly dCT, dA, dC, dT, or dG). Although full-length PrimPol exhibited little affinity for poly dA or poly dC, it bound with significant affinity to poly dT, forming two major intermediates (Figure 3B). This interaction is consistent with PrimPol's preference to catalyze primer synthesis on homopolymeric dT templates (Bianchi et al., 2013). Strikingly, PrimPol formed prominent super-shifted intermediates with poly dG, suggesting that it possesses sequence or structural specificity for dG homopolymers. We repeated the EMSA assays with the catalytic core of the enzyme ($PrimPol_{1-354}$) to determine whether the C-terminal UL52 zinc finger domain, shown previously to bind ssDNA, is required for this preferential homopolymer binding. $PrimPol_{1-354}$ showed a similar profile as the full-length enzyme (Figure S3D), appearing to bind more tightly, suggesting that the AEP catalytic core of the enzyme mainly provides this sequence specificity. Next, to determine whether PrimPol can differentially bind homopolymeric dG sequences flanked by random sequences, and also determine the minimal length of poly dG that can be favorably bound by PrimPol, we repeated the EMSAs with shorter homopolymers located in the middle of random sequence ssDNA. Notably, $PrimPol_{1-354}$ formed stable shifted complexes with short Poly dG (12, 9, and 6 mers), while showing limited affinity for other short 12 nt homopolymers (dA, dC, or dT) (Figure 3C).

Previously, we implicated the repriming activity of PrimPol in maintaining replication fork progression following UV damage in avian cells (Keen et al., 2014). Given the inability of PrimPol to extend through G4 intermediates, we next tested its capacity to reprime on the distal side of these structural barriers. G4#4, which forms a highly stable G4 (Schiavone et al., 2014) and potent block to PrimPol (Figure S3B), was incorporated into a mixed sequence template strand. In order to analyze repriming downstream of the G4, and better represent a situation where replication has stalled at the structure, a primer containing a 3′-dideoxynucleotide (3′-dd) was annealed upstream of the G4 motif (Figure 3D). Additionally, templates containing only a short sequence (5 bases) upstream of the G4, and no 3′-dd primer, were used to eliminate any artifactual results caused by the primer. Although PrimPol was unable to synthesize through this G4 (Figure S3B), it catalyzed de novo synthesis of primer strands on the G4 templates (Figure 3D). The size of the extended products, both on templates with and without a 3′-dd primer, were consistent with repriming ~6 nt downstream of the G4 structure. When tested on the equivalent templates containing no G4 structure, PrimPol synthesized longer and more variable products, suggesting priming in multiple locations further upstream. Based on template configurations and the lengths of fully extended primers, it is apparent that repriming on the G4 templates is occurring almost immediately after the structure, leaving only a minimal sized gap before the restart of replication is resumed. Although PrimPol has preference for primer synthesis on pyrimidine tracts (Bianchi et al., 2013; Rudd et al., 2013), repriming is initiated on these mixed sequence templates. This mechanism is consistent with its proposed role in reinitiating DNA synthesis immediately after G4 and other impediments during replication.

Thus, PrimPol plays a prominent role in preventing fork stalling at G4s in vivo. Although it is unable to directly replicate G4s in vitro, it can bind to the structures and reprime after them. These observations suggested that the repriming activity of PrimPol, dependent on its C-terminal zinc finger, may be its most important contribution to G4 replication in vivo. To test whether both the catalytic and zinc finger domains are needed for the efficient in vivo replication of the +3.5 G4 motif, we complemented *primpol* cells with catalytically inactive human PrimPol (D114A/E116A; AxA) or PrimPol carrying a mutated zinc finger (C419A/H426A; ZfKO). All were fused to YFP to allow expression to be monitored. We then cloned individual YFP-positive Bu-1a^{high} cells, expanded them, and performed a fluctuation analysis for loss of Bu-1a expression. Interestingly, while expression of wild-type PrimPol was stably maintained, expression of both the AxA and ZfKO mutants appeared to be deleterious and was unstable. However, in those cells that retained expression of YFP, which reflected retention of full-length mutant PrimPol (Figure S4), neither mutant was able to prevent instability of Bu-1a expression, in contrast to wild-type PrimPol (Figure 4A). These findings strongly suggest that repriming is the essential mechanism used by PrimPol to ensure stable fork progression at these structural impediments, preventing epigenetic instability caused by formation of long, post-replicative leading strand gaps.

Localization of PrimPol to G4s and its ability to reprime close to them may be facilitated by a combination of the C-terminal domains binding single-stranded DNA and RPA (Guilliam et al., 2015; Wan et al., 2013) and the innate capacity of its catalytic domain to bind homopolymeric sequences and G4 structures (Figure 4B). An interesting outstanding question is whether the G4 binding of PrimPol in vitro also contributes more directly to their bypass in vivo. The avidity of PrimPol for homopolymeric sequences suggests that it could act as a DNA chaperone preventing refolding of G4 motifs into structures during replication fork progression. It is also possible that in vivo PrimPol may act in concert with other factors to melt and stabilize these G4s, in a manner similar to that proposed for RPA and REV1 (Qureshi et al., 2012; Eddy et al., 2014). However, unlike REV1, which appears to be required most at G4s with long loops (Schiavone et al., 2014), the absence of PrimPol is most noticeable at structures of high thermodynamic stability. While this may reflect the stronger binding of PrimPol to more thermodynamically stable G4s, it may also, more simply, result from the intrinsically greater potency of such structures as replication blocks and therefore correlate with the need for repriming. Together with our previous data (Schiavone et al., 2014), the observations presented here provide clear evidence of differential in vivo genetic requirements at G4s with distinct in vitro properties.

EXPERIMENTAL PROCEDURES

DT40 Protocols

DT40 cells were cultured at 37°C in RPMI 1640 with Glutamax supplemented with 7% fetal calf serum and 3% chicken serum. *primpol BU-1*$^{\Delta G4}$ was generated by deleting the +3.5 G4 motif from both alleles of *BU-1* locus, as described

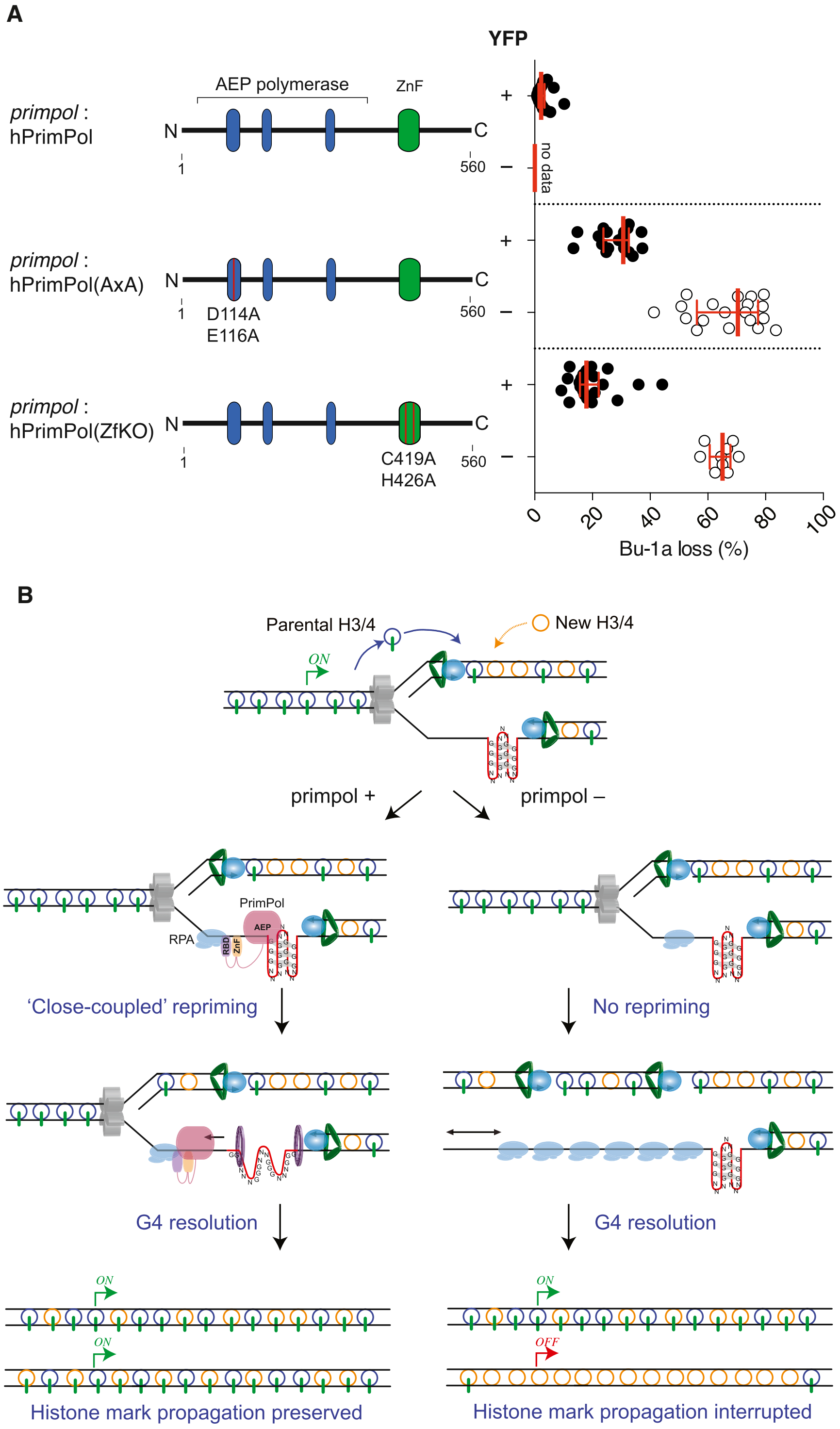

Figure 4. Mechanism of G4 Bypass by PrimPol

(A) Complementation of *primpol* cells with human PrimPol. PrimPol, or mutants, tagged with YFP were expressed in *primpol* cells and single YFP +ve/Bu-1a +ve clones were expanded for 3 weeks. In order to exclude cells that had lost expression of PrimPol during expansion, FACS analysis for Bu-1a expression was performed after gating for YFP expression. The fluctuation analyses are presented separately for YFP +ve (filled circles) and YFP –ve (open circles) cells. Wild-type PrimPol was expressed stably so YFP-ve cells were not generated.

(B) A G4 blocks the leading strand polymerase resulting in exposure initially of a short tract of single-stranded DNA ahead of the G4, to which RPA binds. If PrimPol is present, it can be recruited to the RPA and adjacent ssDNA through its C-terminal RBD and ZnF domains. Binding of the catalytic domain to the G4 may also help localize the enzyme. PrimPol synthesizes a short primer adjacent to the G4 and this allows replication to continue, maintaining coupling of DNA synthesis with the advancing helicase and with histone recycling and thus the parental epigenetic state of the locus is maintained as the cells divide. If PrimPol is absent, there is no repriming resulting in a much longer tract of ssDNA being exposed at the G4. This results in significant displacement of parental histones. The resulting gap may be replicated by a fork arriving from the other direction or by eventual release of the blocked polymerase as the structure is unwound. In either case, if the gap is replicated without the supply of parental histones, the pre-existing epigenetic state is lost. See also Figure S4.

(Schiavone et al., 2014). *BU-1*$^{\Delta G4}$ cells were targeted with PrimPol targeting constructs, as described (Bianchi et al., 2013). G4 knockins in the BU-1 locus performed as described (Schiavone et al., 2014). Complemented cells were obtained by transfecting DT40 PrimPol cells with vectors expressing YFP-tagged PrimPol, the catalytic (AXA) or zinc finger (ZnF) mutants, and selecting puromyci- resistant clones. PrimPol expression was confirmed by flow cytometry (YFP) and western blots (Supplemental Information).

Bu-1a Staining and Fluctuation Analysis for Bu-1a Loss

Confluent cells (0.4 × 10^6 to 2 × 10^6/ml) were directly stained for 10 min with anti-Bu-1a conjugated with phycoerythrin (Santa Cruz clone 5K98) at a 1:10 dilution. Cells analyzed by flow cytometry using an LSRII cytometer. To carry out fluctuation analysis, single cells staining positive for Bu-1a were sorted using a MOFLO sorting cytometer and grown for 20 generations before staining and analysis using flow cytometry as above.

Analysis of Bu1a Locus in PrimPol Bu1a^{low} Cells

A population of *primpol* Bu1a^{low} cells was isolated from a bulk *primpol* population by sorting, see above. Genomic DNA from this population and WT cells was extracted and used for PCR amplification of fragments A, B, and C (Figure S1) using Q5 Hot Start High-Fidelity DNA polymerase (primers are in Supplemental Information). Products cloned into pBluescript, and digested with ApaLI (Fragment A) and ScaI (Fragments B and C). PCR and digestion products run on 0.8% agarose gels. Fragment B cloned from *primpol* Bu1a^{low} cells was also used to make a library in *E. coli*, and the region around the G4 sequenced using primer G4Seq (Supplemental Information) and aligned against the WT sequence.

Chromatin Immunoprecipitation

Chromatin Immunoprecipitation was performed as described (Schiavone et al., 2014) using these antibodies: Histone H3 (Abcam Cat# ab1791 RRID: AB_302613), H3K4me3 (Cell Signaling Technology Cat# 9727L RRID: AB_561097), H3K9me3 (Abcam Cat# ab8898 RRID: AB_306848), and Normal rabbit IgG (Millipore Cat# NI01-100UG RRID: AB_490574). Supplemental Information contains PCR primers for ChIP qPCR.

Bisulphite Treatment and DNA Methylation Mapping

Primpol Bu1a^{high}, Bu1a^{medium}, and Bu1a^{low} pure populations were isolated and genomic DNA extracted. DNA was bisulphite treated using EZ DNA Methylation-Gold Kit. Fragments −0.5 kb and +0.5 kb from the TSS were amplified with ZymoTaq and resulting fragments cloned into pBluescript and sequenced.

Primer Extension Reactions

DNA templates containing G4s were annealed to 5′ hexachlorofluorescein (Hex) labeled primers in annealing buffer plus 50 mM KCl. Primer extension assays were performed using DNA substrates (Supplemental Information). Primer extensions were carried out at 37°C in buffer containing 10 mM Bis-Tris-Propane-HCl (pH 7.0), 50 mM KCl, 10 mM $MgCl_2$, 1 mM DTT, 20 nM DNA, 200 μM dNTPs, and 100 nM PrimPol. Primer extensions were monitored over time (1, 2, 5, 10, and 20 min) and quenched with 200 nM competitor oligo and stop buffer (95% formamide with 0.25% bromophenol blue and xylene cyanol dyes). Products of the reactions were boiled and resolved on a 12.5% (v/v) polyacrylamide/7 M urea gel. Gels scanned using an FLA-5100 image reader (Fujifilm).

Electrophoretic Mobility Shift Assays

Electrophoretic mobility shift assays (EMSAs) were performed in reactions containing 20 mM Tris-HCl (pH 7.5), 30 mM KCl, 1 mM TCEP 0.1 mg/ml BSA, 100 nM Hex-labeled ssDNA, and PrimPol (0.7, 2.5, 5, 10 μM). All ssDNA probes are listed in Supplemental Information. PrimPol and ssDNA were incubated at 25°C for 30 min and then Ficoll was added (2.5% (v/v) final conc.) and resolved on 6% (v/v) native polyacrylamide gels in 0.5 × TBE buffer (50 mM Tris, 50 mM Boric acid, 0.5 mM EDTA). Gels were imaged with a Fuji FLA-5100 imager.

Primase Assays

5′ biotin-labeled DNA was incubated at 95°C in annealing buffer with 50 mM KCl and cooled to allow G4 structure formation and primer annealing. PrimPol (2 μM) was incubated with templates (1 μM) for 30 min at 37°C in buffer (10 mM Bis-Tris-Propane-HCl [pH 7.0], 10 mM $MgCl_2$, 1 mM DTT, 250 μM dNTPs or rNTPs, and 2.5 μM FAM dNTPs). Reactions were quenched with B-W buffer (10 mM Tris-HCl [pH 7.5], 500 mM NaCl, 10 mM EDTA) and incubated with streptavidin beads for 1 hr at 4°C. Beads were washed with B-W buffer and suspended in stop buffer (see above). Samples were heated to 95°C and resolved on a 15% polyacrylamide/7 M urea gel for 90 min. Products were visualized on an FLA-5100 imager.

SUPPLEMENTAL INFORMATION

Supplemental Information includes Supplemental Experimental Procedure and four figures and can be found with this article online at http://dx.doi.org/10.1016/j.molcel.2015.10.038.

AUTHOR CONTRIBUTIONS

D.S. created the *BU-1 primpol* mutants and performed fluctuation analyses. S.K.J. purified full-length and truncated forms of recombinant PrimPol, performed primer extension, and electrophoretic mobility shift assays. M.R. performed the analysis of histone and DNA modifications. G.G. helped design the *BU-1* G4 mutants, identified the three states of *BU-1* expression, and devised the Monte-Carlo simulation of *BU-1* loss. T.A.G. designed and performed the G4 re-priming assays. L.J.B. generated Hs PrimPol wild-type and mutant complimented PrimPol $^{-/-}$ DT40 cell lines. J.E.S. and A.J.D. conceived the project, designed the experiments, and wrote the manuscript.

ACKNOWLEDGMENTS

Work in the A.J.D. laboratory is supported by grants from Biotechnology and Biological Sciences Research Council (BBSRC; BB/H019723/1 and BB/M008800/1). T.A.G. was supported by a U. of Sussex PhD studentship. Work in the J.E.S. lab is supported by a core grant to the MRC to the Laboratory of Molecular Biology (U1051178808). D.S. was supported by an EMBO Long Term Fellowship and M.R. by a Milstein Studentship of the Darwin Trust of Edinburgh.

Received: July 20, 2015
Revised: October 12, 2015
Accepted: October 23, 2015
Published: November 25, 2015

REFERENCES

Bianchi, J., Rudd, S.G., Jozwiakowski, S.K., Bailey, L.J., Soura, V., Taylor, E., Stevanovic, I., Green, A.J., Stracker, T.H., Lindsay, H.D., and Doherty, A.J. (2013). PrimPol bypasses UV photoproducts during eukaryotic chromosomal DNA replication. Mol. Cell *52*, 566–573.

Cheung, I., Schertzer, M., Rose, A., and Lansdorp, P.M. (2002). Disruption of dog-1 in Caenorhabditis elegans triggers deletions upstream of guanine-rich DNA. Nat. Genet. *31*, 405–409.

Eddy, S., Ketkar, A., Zafar, M.K., Maddukuri, L., Choi, J.-Y., and Eoff, R.L. (2014). Human Rev1 polymerase disrupts G-quadruplex DNA. Nucleic Acids Res. *42*, 3272–3285.

García-Gómez, S., Reyes, A., Martínez-Jiménez, M.I., Chocrón, E.S., Mourón, S., Terrados, G., Powell, C., Salido, E., Méndez, J., Holt, I.J., and Blanco, L. (2013). PrimPol, an archaic primase/polymerase operating in human cells. Mol. Cell *52*, 541–553.

Guilliam, T.A., Jozwiakowski, S.K., Ehlinger, A., Barnes, R.P., Rudd, S.G., Bailey, L.J., Skehel, J.M., Eckert, K.A., Chazin, W.J., and Doherty, A.J. (2015). Human PrimPol is a highly error-prone polymerase regulated by single-stranded DNA binding proteins. Nucleic Acids Res. *43*, 1056–1068.

Hardin, C.C., Watson, T., Corregan, M., and Bailey, C. (1992). Cation-dependent transition between the quadruplex and Watson-Crick hairpin forms of d(CGCG3GCG). Biochemistry *31*, 833–841.

Keen, B.A., Jozwiakowski, S.K., Bailey, L.J., Bianchi, J., and Doherty, A.J. (2014). Molecular dissection of the domain architecture and catalytic activities of human PrimPol. Nucleic Acids Res. *42*, 5830–5845.

León-Ortiz, A.M., Svendsen, J., and Boulton, S.J. (2014). Metabolism of DNA secondary structures at the eukaryotic replication fork. DNA Repair (Amst.) *19*, 152–162.

Maizels, N., and Gray, L.T. (2013). The G4 genome. PLoS Genet. *9*, e1003468.

Mourón, S., Rodriguez-Acebes, S., Martínez-Jiménez, M.I., García-Gómez, S., Chocrón, S., Blanco, L., and Méndez, J. (2013). Repriming of DNA synthesis at stalled replication forks by human PrimPol. Nat. Struct. Mol. Biol. *20*, 1383–1389.

Murat, P., and Balasubramanian, S. (2014). Existence and consequences of G-quadruplex structures in DNA. Curr. Opin. Genet. Dev. *25*, 22–29.

Qureshi, M.H., Ray, S., Sewell, A.L., Basu, S., and Balci, H. (2012). Replication protein A unfolds G-quadruplex structures with varying degrees of efficiency. J. Phys. Chem. B *116*, 5588–5594.

Ribeyre, C., Lopes, J., Boulé, J.-B., Piazza, A., Guédin, A., Zakian, V.A., Mergny, J.-L., and Nicolas, A. (2009). The yeast Pif1 helicase prevents genomic instability caused by G-quadruplex-forming CEB1 sequences in vivo. PLoS Genet. *5*, e1000475.

Rudd, S.G., Glover, L., Jozwiakowski, S.K., Horn, D., and Doherty, A.J. (2013). PPL2 translesion polymerase is essential for the completion of chromosomal DNA replication in the African trypanosome. Mol. Cell *52*, 554–565.

Rudd, S.G., Bianchi, J., and Doherty, A.J. (2014). PrimPol—A new polymerase on the block. Mol. Cell. Oncol. *1*, e960754.

Sarkies, P., Reams, C., Simpson, L.J., and Sale, J.E. (2010). Epigenetic instability due to defective replication of structured DNA. Mol. Cell *40*, 703–713.

Sarkies, P., Murat, P., Phillips, L.G., Patel, K.J., Balasubramanian, S., and Sale, J.E. (2012). FANCJ coordinates two pathways that maintain epigenetic stability at G-quadruplex DNA. Nucleic Acids Res. *40*, 1485–1498.

Schiavone, D., Guilbaud, G., Murat, P., Papadopoulou, C., Sarkies, P., Prioleau, M.-N., Balasubramanian, S., and Sale, J.E. (2014). Determinants of G quadruplex-induced epigenetic instability in REV1-deficient cells. EMBO J. *33*, 2507–2520.

Sengar, A., Heddi, B., and Phan, A.T. (2014). Formation of G-quadruplexes in poly-G sequences: structure of a propeller-type parallel-stranded G-quadruplex formed by a G_{15} stretch. Biochemistry *53*, 7718–7723.

Wan, L., Lou, J., Xia, Y., Su, B., Liu, T., Cui, J., Sun, Y., Lou, H., and Huang, J. (2013). hPrimpol1/CCDC111 is a human DNA primase-polymerase required for the maintenance of genome integrity. EMBO Rep. *14*, 1104–1112.

Wickramasinghe, C.M., Arzouk, H., Frey, A., Maiter, A., and Sale, J.E. (2015). Contributions of the specialised DNA polymerases to replication of structured DNA. DNA Repair (Amst.) *29*, 83–90.

Woodford, K.J., Howell, R.M., and Usdin, K. (1994). A novel K(+)-dependent DNA synthesis arrest site in a commonly occurring sequence motif in eukaryotes. J. Biol. Chem. *269*, 27029–27035.

Molecular Cell

Supplemental Information

PrimPol Is Required for Replicative Tolerance of G Quadruplexes in Vertebrate Cells

Davide Schiavone, Stanislaw K. Jozwiakowski, Marina Romanello, Guillaume Guilbaud, Thomas A. Guilliam, Laura J. Bailey, Julian E. Sale, and Aidan J. Doherty

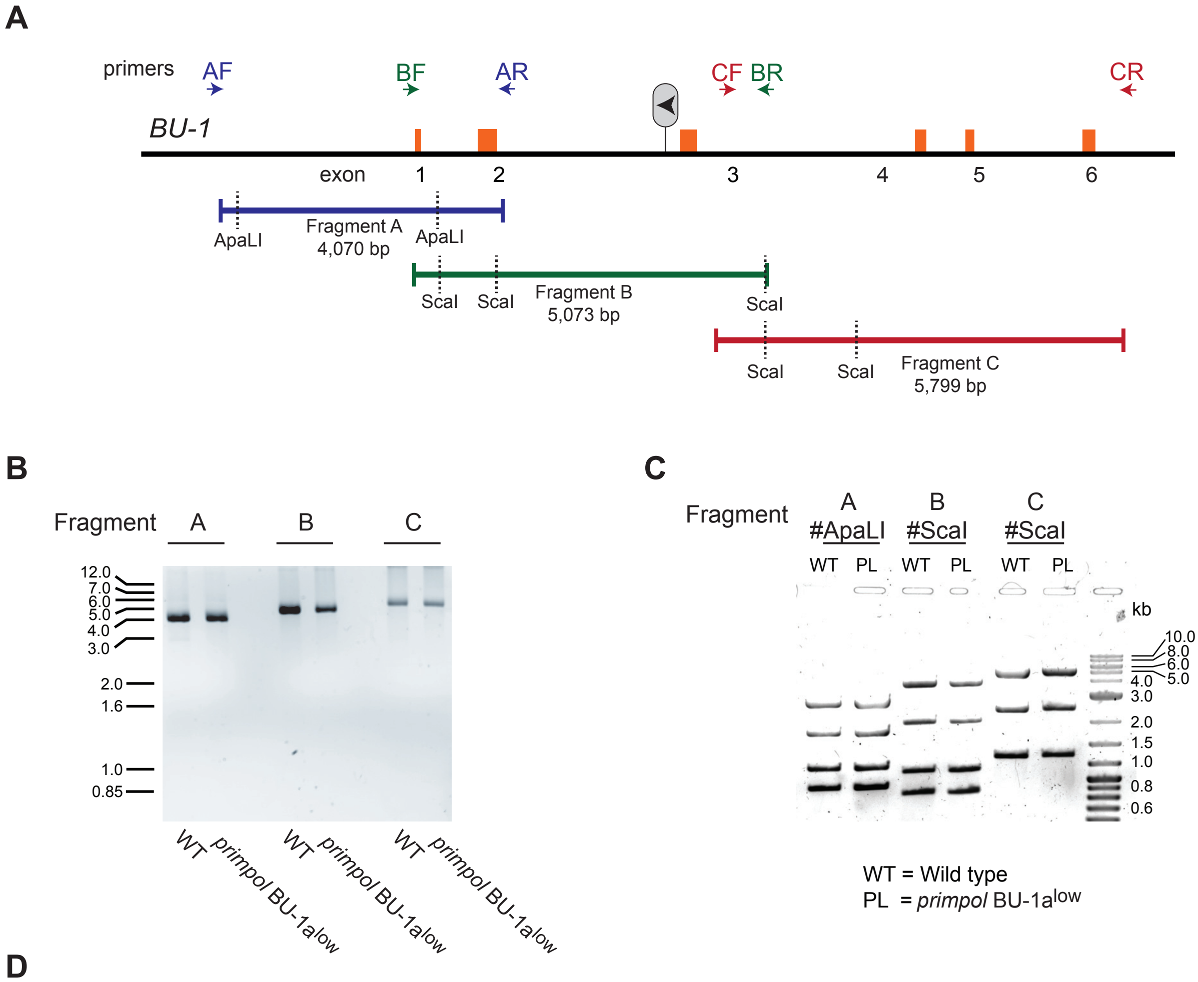

D

```
DT40 Bu1a          1 GTGTCTGGCGTTGACACCTACCTTGAGCATGGTTAGTACCAATTCCTCTCTAAAACAGAAGCAGATACAGTAACAGCAAAAGTACAGACCTCTGGCCAACGAGAAGGCTGACTCTCCTCTGAAGCTATAA 130
Primpol Bu1 low    1 GTGTCTGGCGTTGACACCTACCTTGAGCATGGTTAGTACCAATTCCTCTCTAAAACAGAAGCAGATACAGTAACAGCAAAAGTACAGACCTCTGGCCAACGAGAAGGCTGACTCTCCTCTGAAGCTATAA 130
Primpol Bu1 low    1 GTGTCTGGCGTTGACACCTACCTTGAGCATGGTTAGTACCAATTCCTCTCTAAAACAGAAGCAGATACAGTAACAGCAAAAGTACAGACCTCTGGCCAACGAGAAGGCTGACTCTCCTCTGAAGCTATAA 130
Primpol Bu1 low    1 GTGTCTGGCGTTGACACCTACCTTGAGCATGGTTAGTACCAATTCCTCTCTAAAACAGAAGCAGATACAGTAACAGCAAAAGTACAGACCTCTGGCCAACGAGAAGGCTGACTCTCCTCTGAAGCTATAA 130
Primpol Bu1 low    1 GTGTCTGGCGTTGACACCTACCTTGAGCATGGTTAGTACCAATTCCTCTCTAAAACAGAAGCAGATACAGTAACAGCAAAAGTACAGACCTCTGGCCAACGAGAAGGCTGACTCTCCTCTGAAGCTATAA 130
Primpol Bu1 low    1 GTGTCTGGCGTTGACACCTACCTTGAGCATGGTTAGTACCAATTCCTCTCTAAAACAGAAGCAGATACAGTAACAGCAAAAGTACAGACCTCTGGCCAACGAGAAGGCTGACTCTCCTCTGAAGCTATAA 130
Primpol Bu1 low    1 GTGTCTGGCGTTGACACCTACCTTGAGCATGGTTAGTACCAATTCCTCTCTAAAACAGAAGCAGATACAGTAACAGCAAAAGTACAGACCTCTGGCCAACGAGAAGGCTGACTCTCCTCTGAAGCTATAA 130
Primpol Bu1 low    1 GTGTCTGGCGTTGACACCTACCTTGAGCATGGTTAGTACCAATTCCTCTCTAAAACAGAAGCAGATACAGTAACAGCAAAAGTACAGACCTCTGGCCAACCAGAAGGCTGACTCTCCTCTGAAGCTATAA 130
Primpol Bu1 low    1 GTGTCTGGCGTTGACACCTACCTTGAGCATGGTTAGTACCAATTCCTCTCTAAAACAGAAGCAGATACAGTAACAGCAAAAGTACAGACCTCTGGCCAACCAGAAGGCTGACTCTCCTCTGAAGCTATAA 130
Primpol Bu1 low    1 GTGTCTGGCGTTGACACCTACCTTGAGCATGGTTAGTACCAATTCCTCTCTAAAACAGAAGCAGATACAGTAACAGCAAAAGTACAGACCTCTGGCCAACCAGAAGGCTGACTCTCCTCTGAAGCTATAA 130
                     **************************************************************************************************** *****************************

DT40 Bu1a        131 TGGGTATAATCAATTACCTATTCATTATATAGGAAGCCTCTGGCCAGCAGAAGCTTAGCGTGCCCTCAGATGATGGTCTTCTGTTGCTATCATTTTCACTTTCAACAAGGAAAATAAAGAGGTGCCATAT 260
Primpol Bu1 low  131 TGGGTATAATCAATTACCTATTCATTATATAGGAAGCCTCTGGCCAGCAGAAGCTTAGCGTGCCCTCAGATGATGGTCTTCTGTTGCTATCATTTTCACTTTCAACAAGGAAAATAAAGAGGTGCCATAT 260
Primpol Bu1 low  131 TGGGTATAATCAATTACCTATTCATTATATAGGAAGCCTCTGGCCAGCAGAAGCTTAGCGTGCCCTCAGATGATGGTCTTCTGTTGCTATCATTTTCACTTTCAACAAGGAAAATAAAGAGGTGCCATAT 260
Primpol Bu1 low  131 TGGGTATAATCAATTACCTATTCATTATATAGGAAGCCTCTGGCCAGCAGAAGCTTAGCGTGCCCTCAGATGATGGTCTTCTGTTGCTATCATTTTCACTTTCAACAAGGAAAATAAAGAGGTGCCATAT 260
Primpol Bu1 low  131 TGGGTATAATCAATTACCTATTCATTATATAGGAAGCCTCTGGCCAGCAGAAGCTTAGCGTGCCCTCAGATGATGGTCTTCTGTTGCTATCATTTTCACTTTCAACAAGGAAAATAAAGAGGTGCCATAT 260
Primpol Bu1 low  131 TGGGTATAATCAATTACCTATTCATTATATAGGAAGCCTCTGGCCAGCAGAAGCTTAGCGTGCCCTCAGATGATGGTCTTCTGTTGCTATCATTTTCACTTTCAACAAGGAAAATAAAGAGGTGCCATAT 260
Primpol Bu1 low  131 TGGGTATAATCAATTACCTATTCATTATATAGGAAGCCTCTGGCCAGCAGAAGCTTAGCGTGCCCTCAGATGATGGTCTTCTGTTGCTATCATTTTCACTTTCAACAAGGAAAATAAAGAGGTGCCATAT 260
Primpol Bu1 low  131 TGGGTATAATCAATTACCTATTCATTATATAGGAAGCCTCTGGCCAGCAGAAGCTTAGCGTGCCCTCAGATGATGGTCTTCTGTTGCTATCATTTTCACTTTCAACAAGGAAAATAAAGAGGTGCCATAT 260
Primpol Bu1 low  131 TGGGTATAATCAATTACCTATTCATTATATAGGAAGCCTCTGGCCAGCAGAAGCTTAGCGTGCCCTCAGATGATGGTCTTCTGTTGCTATCATTTTCACTTTCAACAAGGAAAATAAAGAGGTGCCATAT 260
Primpol Bu1 low  131 TGGGTATAATCAATTACCTATTCATTATATAGGAAGCCTCTGGCCAGCAGAAGCTTAGCGTGCCCTCAGATGATGGTCTTCTGTTGCTATCATTTTCACTTTCAACAAGGAAAATAAAGAGGTGCCATAT 260
                     **********************************************************************************************************************************

DT40 Bu1a        261 TTTTAGGGTGTCCCCAAAATGTTGACCAAGTGTCACCAGTAGGGATGGGTGGGTGCTGTCAAGGGCTGGGATCTAGCAATTGAAGAGTCTGGTTACTTAGTGATGTGCTCCCTGGCTAACAGTGAATGTG 390
Primpol Bu1 low  261 TTTTAGGGTGTCCCCAAAATGTTGACCAAGTGTCACCAGTAGGGATGGGTGGGTGCTGTCAAGGGCTGGGATCTAGCAATTGAAGAGTCTGGTTACTTAGTGATGTGCTCCCTGGCTAACAGTGAATGTG 390
Primpol Bu1 low  261 TTTTAGGGTGTCCCCAAAATGTTGACCAAGTGTCACCAGTAGGGATGGGTGGGTGCTGTCAAGGGCTGGGATCTAGCAATTGAAGAGTCTGGTTACTTAGTGATGTGCTCCCTGGCTAACAGTGAATGTG 390
Primpol Bu1 low  261 TTTTAGGGTGTCCCCAAAATGTTGACCAAGTGTCACCAGTAGGGATGGGTGGGTGCTGTCAAGGGCTGGGATCTAGCAATTGAAGAGTCTGGTTACTTAGTGATGTGCTCCCTGGCTAACAGTGAATGTG 390
Primpol Bu1 low  261 TTTTAGGGTGTCCCCAAAATGTTGACCAAGTGTCACCAGTAGGGATGGGTGGGTGCTGTCAAGGGCTGGGATCTAGCAATTGAAGAGTCTGGTTACTTAGTGATGTGCTCCCTGGCTAACAGTGAATGTG 390
Primpol Bu1 low  261 TTTTAGGGTGTCCCCAAAATGTTGACCAAGTGTCACCAGTAGGGATGGGTGGGTGCTGTCAAGGGCTGGGATCTAGCAATTGAAGAGTCTGGTTACTTAGTGATGTGCTCCCTGGCTAACAGTGAATGTG 390
Primpol Bu1 low  261 TTTTAGGGTGTCCCCAAAATGTTGACCAAGTGTCACCAGTAGGGATGGGTGGGTGCTGTCAAGGGCTGGGATCTAGCAATTGAAGAGTCTGGTTACTTAGTGATGTGCTCCCTGGCTAACAGTGAATGTG 390
Primpol Bu1 low  261 TTTTAGGGTGTCCCCAAAATGTTGACCAAGTGTCACCAGTAGGGATGGGTGGGTGCTGTCAAGGGCTGGGATCTAGCAATTGAAGAGTCTGGTTACTTAGTGATGTGCTCCCTGGCTAACAGTGAATGTG 390
Primpol Bu1 low  261 TTTTAGGGTGTCCCCAAAATGTTGACCAAGTGTCACCAGTAGGGATGGGTGGGTGCTGTCAAGGGCTGGGATCTAGCAATTGAAGAGTCTGGTTACTTAGTGATGTGCTCCCTGGCTAACAGTGAATGTG 390
Primpol Bu1 low  261 TTTTAGGGTGTCCCCAAAATGTTGACCAAGTGTCACCAGTAGGGATGGGTGGGTGCTGTCAAGGGCTGGGATCTAGCAATTGAAGAGTCTGGTTACTTAGTGATGTGCTCCCTGGCTAACAGTGAATGTG 390
                     **********************************************************************************************************************************
                                                             +3.5 G4

DT40 Bu1a        391 GTGGGAGCCCTCCTGGTAGCGGGGAGCTCTCAGTTACTGCTGAGTCTGCTCATCATCTCTCTTCCCTCTCCATTCCCCATTGCCCTGTGCTACGTGACCAGAGCCGATCATTGATGCCCGG 511
Primpol Bu1 low  391 GTGGGAGCCCTCCTGGTAGCGGGGAGCTCTCAGTTACTGCTGAGTCTGCTCATCATCTCTCTTCCCTCTCCATTCCCCATTGCCCTGTGCTACGTGACCAGAGCCGATCATTGATGCCCGG 511
Primpol Bu1 low  391 GTGGGAGCCCTCCTGGTAGCGGGGAGCTCTCAGTTACTGCTGAGTCTGCTCATCATCTCTCTTCCCTCTCCATTCCCCATTGCCCTGTGCTACGTGACCAGAGCCGATCATTGATGCCCGG 511
Primpol Bu1 low  391 GTGGGAGCCCTCCTGGTAGCGGGGAGCTCTCAGTTACTGCTGAGTCTGCTCATCATCTCTCTTCCCTCTCCATTCCCCATTGCCCTGTGCTACGTGACCAGAGCCGATCATTGATGCCCGG 511
Primpol Bu1 low  391 GTGGGAGCCCTCCTGGTAGCGGGGAGCTCTCAGTTACTGCTGAGTCTGCTCATCATCTCTCTTCCCTCTCCATTCCCCATTGCCCTGTGCTACGTGACCAGAGCCGATCATTGATGCCCGG 511
Primpol Bu1 low  391 GTGGGAGCCCTCCTGGTAGCGGGGAGCTCTCAGTTACTGCTGAGTCTGCTCATCATCTCTCTTCCCTCTCCATTCCCCATTGCCCTGTGCTACGTGACCAGAGCCGATCATTGATGCCCGG 511
Primpol Bu1 low  391 GTGGGAGCCCTCCTGGTAGCGGGGAGCTCTCAGTTACTGCTGAGTCTGCTCATCATCTCTCTTCCCTCTCCATTCCCCATTGCCCTGTGCTACGTGACCAGAGCCGATCATTGATGCCCGG 511
Primpol Bu1 low  391 GTGGGAGCCCTCCTGGTAGCGGGGAGCTCTCAGTTACTGCTGAGTCTGCTCATCATCTCTCTTCCCTCTCCATTCCCCATTGCCCTGTGCTACGTGACCAGAGCCGATCATTGATGCCCGG 511
Primpol Bu1 low  391 GTGGGAGCCCTCCTGGTAGCGGGGAGCTCTCAGTTACTGCTGAGTCTGCTCATCATCTCTCTTCCCTCTCCATTCCCCATTGCCCTGTGCTACGTGACCAGAGCCGATCATTGATGCCCGG 511
Primpol Bu1 low  391 GTGGGAGCCCTCCTGGTAGCGGGGAGCTCTCAGTTACTGCTGAGTCTGCTCATCATCTCTCTTCCCTCTCCATTCCCCATTGCCCTGTGCTACGTGACCAGAGCCGATCATTGATGCCCGG 511
                     *************************************************************************************************************************
```

Supplementary Figures

Figure S1. No evidence of high levels of mutation or genetic instability in the *BU-1* locus of Bu-1a^{low} cells. Related to Figure 1.

A. Diagram of the *BU-1* locus indicating PCR fragments A, B & C used to detect gross sequence deletions and the relevant restriction enzyme cutting sites in the fragments.

B. PCR amplification of fragments A, B & C from wild type cells and a pool of sorted Bu-1a^{low} cells.

C. Restriction digestion of the fragments shown in (B) cloned into pBluescript.

D. Sequences from 9 clones taken from around the +3.5 G4 (highlighted in bold). A polymorphism identifies the *BU-1A* allele (red) and *BU-1B* allele (blue).

Schiavone, Jozwiakowski et al. Figure S2

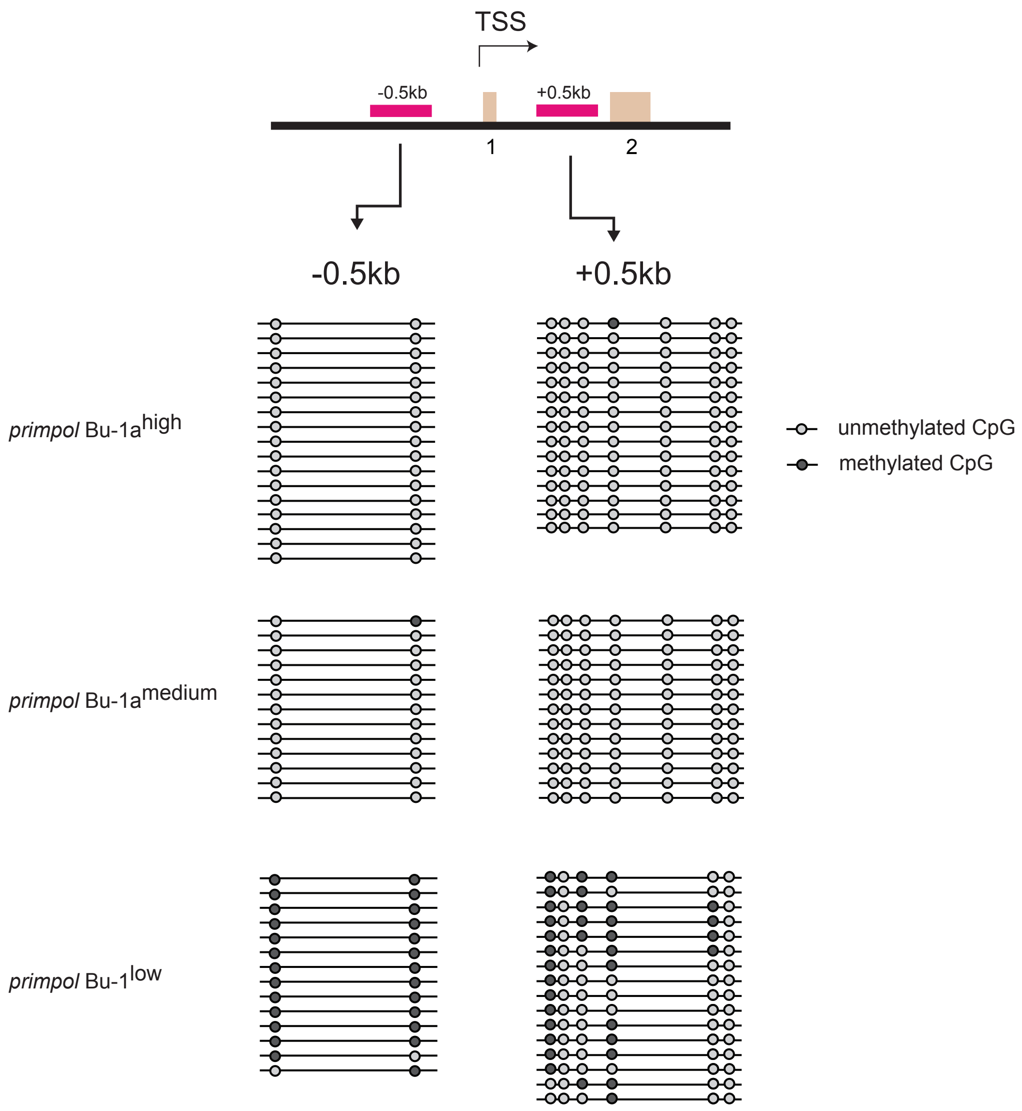

Figure S2. DNA methylation of the *BU-1* promoter determined by bisulphite sequencing. Related to Figure 1.

Bisulphite sequencing results for primers spanning regions located approximately 0.5 kb up and downstream of the TSS of *BU-1*. CpG sites are indicated as circles. Open circles = unmethylated; filled circles = methylated.

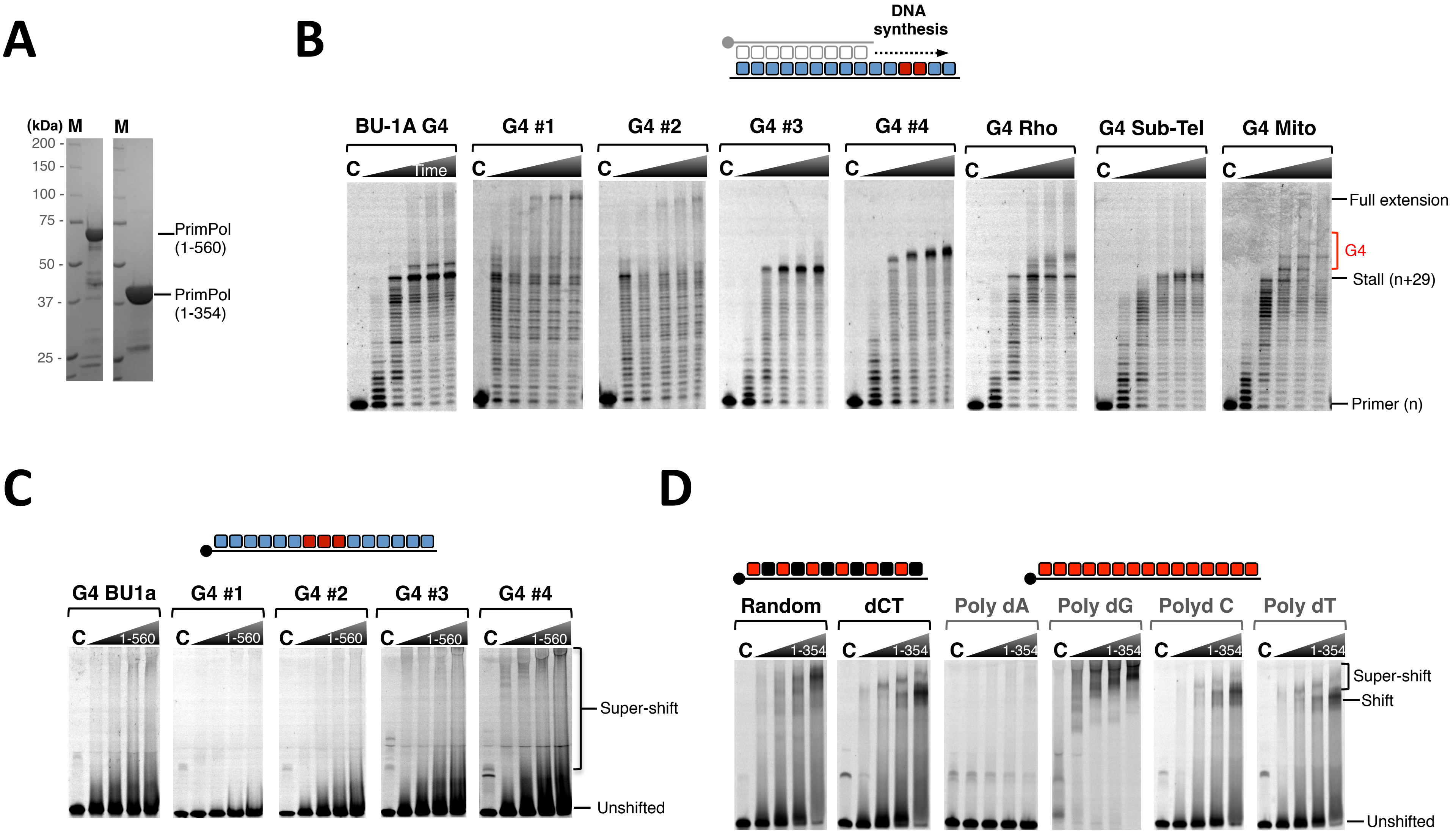
A
(kDa) M
M
200 -
150 -
100 -
75 -
50 -
37 -
25 -
PrimPol (1-560)
PrimPol (1-354)
B
DNA synthesis
BU-1A G4
G4 #1
G4 #2
G4 #3
G4 #4
G4 Rho
G4 Sub-Tel
G4 Mito
C
Time
Full extension
G4
Stall (n+29)
Primer (n)
C
G4 BU1a
G4 #1
G4 #2
G4 #3
G4 #4
1-560
Super-shift
Unshifted
D
Random
dCT
Poly dA
Poly dG
Polyd C
Poly dT
1-354
Super-shift
Shift
Unshifted

Figure S3. Preparation of recombinant human PrimPol proteins, primer extensions and electrophoretic mobility shift assays. Related to Figure 3.

A. SDS PAGE analysis of human recombinant PrimPol proteins. Histidine-tagged full-length human PrimPol (1-560) was purified using Ni-NTA affinity purification, heparin, and size-exclusion chromatography. Glutathione S-transferase (GST) tagged truncation of human PrimPol (1-354) was purified using Glutathione affinity chromatography. The GST tag was cleaved off by incubation with prescission protease over night at 4°C. Subsequently, the tagless truncation of PrimPol (1-354) was purified by heparin and size-exclusion chromatography.

B. Histidine-tagged full length PrimPol (1-560) at 100 nM was incubated with 20 nM G4 quadruplex containing substrates and 200 μM dNTPs for increasing times (1, 2, 5, 10, 20 minutes) at 37°C. A no enzyme control ("C") was performed as a single 20 minute time point at 37°C in the absence of PrimPol.

C. Increasing concentrations (0.7, 2.5, 5, 10 μM) of $\text{PrimPol}_{1\text{-}560}$ were incubated with 100 nM fluorescently labelled ssDNA probes. A no enzyme control ("C") was also performed to monitor mobility of ssDNA probes. The G4 quadruplex structures are marked as red boxes.

D. Increasing concentrations (0.7, 2.5, 5, 10 μM) of $\text{PrimPol}_{1\text{-}354}$ were incubated with 100 nM fluorescently labelled ssDNA probes. A no enzyme control ("C") was also performed to monitor the mobility of ssDNA probes. The homopolymeric nucleotide runs are marked as red boxes and mixed sequences as red and black boxes.

Schiavone, Jozwiakowski et al. Figure S4

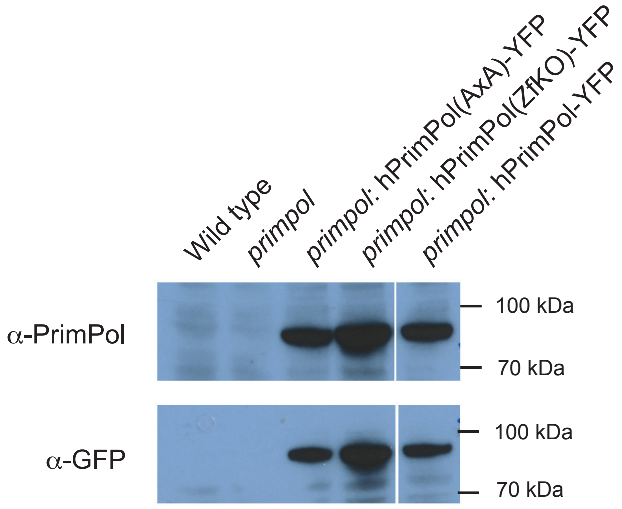

Figure S4. Western blot of YFP-tagged human PrimPol and mutant derivatives in *primpol* DT40 cells. Related to Figure 4.

Whole cell lysates blotted with anti-human PrimPol and anti-GFP. Note the anti-human PrimPol antibody does not cross-react against the chicken protein.

Supplemental Experimental Procedures

Primers for ChIP qPCR

(*c.* -0.5kb and +0.5kb from the *BU-1* TSS)

ChIP -0.5 F	TAGCTCCAAGGTGTGGGACTTT
ChIP -0.5 R	CCCCATACTGGACAGACTGAATA
ChIP +0.5 F	GGCAGCTCAGCAAAGTTTCC
ChIP +0.5 R	GACCACAGCCGTGGAACAGTTA

Primers for methylation analysis

(*c.* -0.5kb and +0.5kb from the *BU-1* TSS)

Methyl -0.5 F	GTTTCTT**GAGCTC**TTTGGTAAGTGATAGTTATTGGTATTGTA
Methyl -0.5 R	GTTTCTT**GCGGCCGC**ATTAACTAAACTCAAACTAAACCAACAC
Methyl +0.5 F	GTTTCTT**GAGCTC**GAATATAAGGTTTTGGTATGTAGAATGT
Methyl +0.5 R	GTTTCTT**GCGGCCGC**CTCCCTAATCACTAAAATTATATACAAAA

Colour code: (Pig tail) SacI/**NotI**

PCR primers for hPrimPol

Amplicon	Forward Primer (5’→3’)	Reverse Primer (5’→3’)
hPrimPol$_{1\text{-}354}$	GTTTCTTGGATCCATGAATA GAAAATGGGAAGCAAAAC	CTTTGTTGCGGCCGCTTACTCTTGTAATACTTCTATAATT AGTTCATCAGGAATTTC

Synthetic primer-template substrates

Figure (Substrate)	Primer (5’→3’)	Template (5’→3’)
S3B (G4 BU1a)	TGTCGTCTGTTCGGTC GTTC	ACCGCGAACTTGAATTCTAGGGCTGGGTGGGTGCTGT CAAGGGCTGGGCAATGCACAACATATGGCTTTCGAAG ACCGAACGACCGAACAGACGACA
S3B (G4#1)	TGTCGTCTGTTCGGTC GTTC	ACCGCGAACTTGAATTCTATTGGTTTTGGTTTTGGTTTT GGTCAATGCACAACATATGGCTTTCGAAGACCGAACGA CCGAACAGACGACA
S3B (G4#2)	TGTCGTCTGTTCGGTC GTTC	ACCGCGAACTTGAATTCTATGGGTTTGGGTTTGGGTTT GGGTCAATGCACAACATATGGCTTTCGAAGACCGAAC GACCGAACAGACGACA
S3B (G4#3)	TGTCGTCTGTTCGGTC GTTC	ACCGCGAACTTGAATTCTATGGGGTTGGGGTTGGGGT TGGGGCAATGCACAACATATGGCTTTCGAAGACCGAA CGACCGAACAGACGACA
S3B (G4#4)	TGTCGTCTGTTCGGTC GTTC	ACCGCGAACTTGAATTCTATTTTGGGTGGGTGGGTGG GTTTTCAATGCACAACATATGGCTTTCGAAGACCGAAC GACCGAACAGACGACA
S3B (G4 Rho)	TGTCGTCTGTTCGGTC GTTC	ACCGCGAACTTGAATTCTAGGGAGTAAAAGGGAGCG GGTGCTGGGGCAATGCACAACATATGGCTTTCGAAGA CCGAACGACCGAACAGACGACA

S3B (G4 Sub-Tel)	TGTCGTCTGTTCGGTC GTTC	ACCGCGAACTTGAATTCTATTAGGGTTAGGGTTAGGGTTAGGGCAATGCACAACATATGGCTTTCGAAGACCGAACGACCGAACAGACGACA
S3B (G4 Mito)	TGTCGTCTGTTCGGTC GTTC	ACCGCGAACTTGAATTCTAGGGTGGGATGGGCGGGGGCAATGCACAACATATGGCTTTCGAAGACCGAACGACCGAACAGACGACA

Synthetic ssDNA probes

**Figure (Substrate)**	**ssDNA probe (5'→3')**
F3A (G4 BU1a)	CTTGAATTCTAGGGCTGGGTGGGTGCTGTCAAGGGCTGGGCACATATGTTC
F3A (G4#1)	CTTGAATTCTATTGGTTTTGGTTTTGGTTTTGGTCACATATGTTC
F3A (G4#2)	CTTGAATTCTATGGGTTTGGGTTTGGGTTTGGGTCACATATGTTC
F3A (G4#3)	CTTGAATTCTATGGGGTTGGGGTTGGGGTTGGGGCACATATGTTC
F3A (G4#4)	CTTGAATTCTATTTTGGGTGGGTGGGTGGGTTTTCACATATGTTC
F3B (Random)	CGCGCAGGGCGCACAACAGCCTTGAAGACCGAACGACCGAACAGACGACA
F3B (dCT)	CTCTCTCTCTCTCTCTCTCTCTCTCTCTCTCTCTCTCTCTCTCTCTCT
F3B (Poly dA)	AAAAAAAAAAAAAAAAAAAAAAAAAAAAAAAAAAAAAAAAAAAAAAAA
F3B (Poly dC)	CCCCCCCCCCCCCCCCCCCCCCCCCCCCCCCCCCCCCCCCCCCCCCCC
F3B (Poly dT)	TTTTTTTTTTTTTTTTTTTTTTTTTTTTTTTTTTTTTTTTTTTTTTTT
F3B (Poly dG)	GGGGGGGGGGGGGGGGGGGGGGGGGGGGGGGGGGGGGGGGGGGGGGGG
F3C (dA12)	CTTGAATTCTT**AAAAAAAAAAAA**TCGTAGCAGTACACATATGTTC
F3C (dC12)	CTTGAATTCTA**CCCCCCCCCCCC**ACGTAGCAGTACACATATGTTC
F3C (dT12)	CTTGAATTCTA**TTTTTTTTTTTT**ACGTAGCAGTACACATATGTTC
F3C (dG12)	CTTGAATTCTA**GGGGGGGGGGGG**ACGTAGCAGTACACATATGTTC
F3C (dG9)	CTTGAATTCTAAC**GGGGGGGGG**TACGTAGCAGTACACATATGTTC
F3C (dG6)	CTTGAATTCTAACA**GGGGGG**TATACGTAGCAGTACACATATGTTC
S3C (G4 BU1a)	CTTGAATTCTAGGGCTGGGTGGGTGCTGTCAAGGGCTGGGCACATATGTTC
S3C (G4#1)	CTTGAATTCTATTGGTTTTGGTTTTGGTTTTGGTCACATATGTTC
S3C (G4#2)	CTTGAATTCTATGGGTTTGGGTTTGGGTTTGGGTCACATATGTTC
S3C (G4#3)	CTTGAATTCTATGGGGTTGGGGTTGGGGTTGGGGCACATATGTTC
S3C (G4#4)	CTTGAATTCTATTTTGGGTGGGTGGGTGGGTTTTCACATATGTTC

S3D (Random)	CGCGCAGGGCGCACAACAGCCTTGAAGACCGAACGACCGAACAGACGACA
S3D (dCT)	CTCTCTCTCTCTCTCTCTCTCTCTCTCTCTCTCTCTCTCTCTCTCTCTCT
S3D (Poly dA)	AAAAAAAAAAAAAAAAAAAAAAAAAAAAAAAAAAAAAAAAAAAAAAAAAA
S3D (Poly dG)	GGGGGGGGGGGGGGGGGGGGGGGGGGGGGGGGGGGGGGGGGGGGGGGGGG
S3D (Poly dC)	CCCCCCCCCCCCCCCCCCCCCCCCCCCCCCCCCCCCCCCCCCCCCCCCCC
S3D (Poly dT)	TTTTTTTTTTTTTTTTTTTTTTTTTTTTTTTTTTTTTTTTTTTTTTTTTT

Primers for BU-1 amplification and G4 locus sequencing

(*Fragments A, B and C*)

Fragm_A_F	GTTTCTTGCGGCCGCTCGATCCTTATGGGTCCCTTCTA
Fragm_A_R	GTTTCTTCTCGAGGCTCCTAAAAGCTCAAGAGGTCT
Fragm_B_F	GTTTCTTGCGGCCGCCTCTGTAGCCAGATCGTCTTCTC
Fragm_B_R	GTTTCTTCTCGAGTTCATACCAACTCTACACCAGCC
Fragm_C_F	GTTTCTTGCGGCCGCGACATTCACCCATAACCCTGACT
Fragm_C_R	GTTTCTTCTCGAGGAGATCTCTGGTGGTTCCTTGTT
G4seq	GTAGGAAGTAGGCTGGTTCCTTGC

Pigtail NotI/XhoI

Primase assay substrates

**Figure (Substrate)**	**Primer (5'→3')**	**Template (5'→3')**
F3D (G4, left)	GATGCATCTATGTddG	GTCTTCTATCTCGTCTATATTCTATTGTCTCTATGGGTG GGTGGGTGGGTCTCACATAGATGCATC
F3D (Control, left)	GATGCATCTATGTddG	GTCTTCTATCTCGTCTATATTCTATTGTCTCTATGAATA CCTTCATCAGTCTCACATAGATGCATC
F3D (G4, right)	N/A	GTCTTCTATCTCGTCTATATTCTATTGTCTCTATGGGTG GGTGGGTGGGTCTCA
F3D (Control, right)	N/A	GTCTTCTATCTCGTCTATATTCTATTGTCTCTATGAATA CCTTCATCAGTCTCACATAGATGCATC
